# Identifying and embedding transferability in data-driven representations of chemical space†

**DOI:** 10.1039/d4sc02358g

**Published:** 2024-06-21

**Authors:** Tim Gould, Bun Chan, Stephen G. Dale, Stefan Vuckovic

**Affiliations:** a Queensland Micro- and Nanotechnology Centre, Griffith University Nathan Qld 4111 Australia; b Graduate School of Engineering, Nagasaki University Bunkyo 1-14 Nagasaki 852-8521 Japan; c Institute of Functional Intelligent Materials, National University of Singapore 4 Science Drive 2 Singapore 117544; d Department of Chemistry, University of Fribourg Fribourg Switzerland stefan.vuckovic@unifr.ch

## Abstract

Transferability, especially in the context of model generalization, is a paradigm of all scientific disciplines. However, the rapid advancement of machine learned model development threatens this paradigm, as it can be difficult to understand how transferability is embedded (or missed) in complex models developed using large training data sets. Two related open problems are how to identify, without relying on human intuition, what makes training data transferable; and how to embed transferability into training data. To solve both problems for *ab initio* chemical modelling, an indispensable tool in everyday chemistry research, we introduce a transferability assessment tool (TAT) and demonstrate it on a controllable data-driven model for developing density functional approximations (DFAs). We reveal that human intuition in the curation of training data introduces chemical biases that can hamper the transferability of data-driven DFAs. We use our TAT to motivate three transferability principles; one of which introduces the key concept of transferable diversity. Finally, we propose data curation strategies for general-purpose machine learning models in chemistry that identify and embed the transferability principles.

## Abstract 2

We show that human intuition in the curation of training data introduces biases that hamper model transferability. We introduce a transferability assessment tool which rigorously measures and subsequently improves transferability.

## Graphical abstract

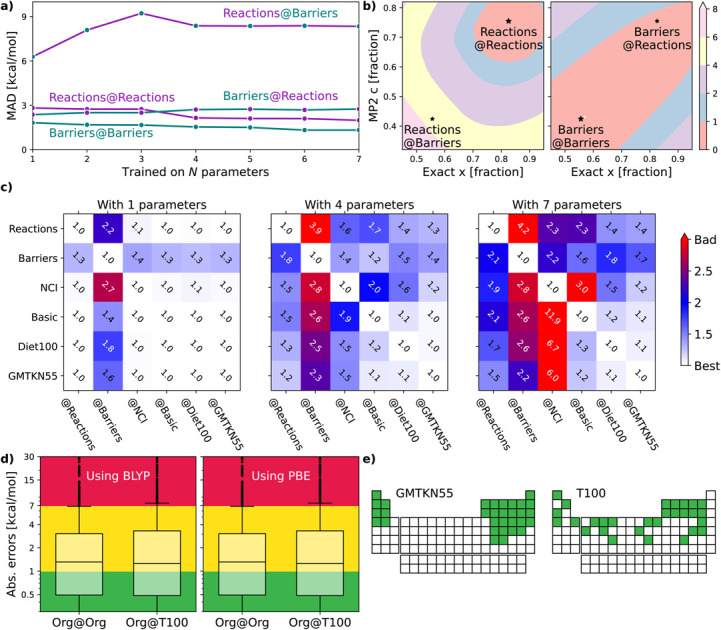

## 1. Introduction

For the past half-century, Density Functional Theory (DFT)^1,2^ has made an unparalleled impact across a range of scientific and engineering disciplines. Nowadays, this impact is greater than ever, as evidenced by the large portion of the world's supercomputing power being consumed by DFT simulations.^3,4^ In recent years, machine learning (ML) is transforming nearly all scientific disciplines, and DFT is no exception.^5,6^ The use of DFT in tandem with statistical learning is ever growing,^7–11^ and recent advancements in ML-based DFT^12^ signal the beginning of a race to discover the DFT ‘holy grail’ or at least a highly effective surrogate thereof – holding promise to revolutionize the entire field of chemistry.^13^ Building on this momentum, ML of density functional approximations (DFAs) is enabling rapid advances in the predictive quality of quantum chemistry, by enhancing the practical cost and quality benefits of DFT by empirical strategies based on “big data” training sets.^14,15^

The assumption that a DFA is transferable is implicit in every DFA developed for general use, and this culture of universal density functionals has been readily adopted by the machine-learned DFA (ML-DFA) community. While it has long been understood that DFAs tend to perform better on some chemistries (*e.g.* ‘typical’ organic bonds), and worse on others (*e.g.* transition metal bonds), the very nature of data-driven development (*e.g.* for ML-DFAs or empirical DFAs) more heavily weights performance on training sets, whereas the traditional strategy tends to rely more on universal limits like homogeneous electron gases that are less likely to bias to specific realistic systems. There is thus an urgent need to understand how transferability is embedded in training data, so that ML-DFAs developed using the training data can be relied upon to extrapolate (transfer) to new systems outside the training data and any initial tests – something that is demonstrably not guaranteed in ML-DFAs.^17^ Understanding how to embed transferability first requires an understanding of how to identify transferability.

To solve both these problems, this work will introduce a transferability assessment tool that involves training DFAs on a test set **A**, and assessing the performance of that functional on test set **B**, abbreviated to **B@A** (or [test set]@[training set]), more details given in Section 2. Achieving high performance on **A@A** is often straightforward, as we can always increase model flexibility by adding more parameters. However, the true challenge lies in ensuring that the (ML-)DFA is transferable to **B** (*i.e.***B@A**), meaning it genuinely learns (and may thus extrapolate) rather than simply memorizes patterns in A. This task prompts a range of questions.

**(1)** First, a key and outstanding problem is how do we create **A** to embed transferability of our ML-DFA model to a wide range of chemical physics?

**(2)** Is more always more (*i.e.* does increasing the size of set **A** always improve **B@A**?)

**(3)** Can we quantify how difficult test set **B** is for a model trained on **A**? (*e.g.* can we quantify the intuition that training a model on atomisation energies of alkanes better predicts atomisation energies of alkenes than transition metal barrier heights?)

**(4)** Can we quantify the ‘distance’ or difficulty level between training set **A** and test set **B**?

**(5)** Does the inclusion of well-known or well-studied chemical structures in **A** enhance or limit the model's transferability to unseen chemistry?

After all, the ultimate goal of DFT simulations is not just to confirm and rationalize what we already know from experiments but to accurately predict (transfer to) unseen chemistry and unperformed experiments.^13^

In using the transferability assessment tool (TAT) to explore the above questions, we show that simply expanding the number and/or type of chemical systems in a given training set is insufficient to improve an ML-DFA in general (Section 3). By contrast, we reveal three transferability principles that do embed transferability in a benchmark set (benchset for brevity), taken together, and that may therefore be used in the curation of better training benchsets. Most importantly, we introduce the concept of transferable diversity to our training set design – meaning we aim for our training set to yield good transferability to a diverse range of chemical behaviours. We use these principles to design the **T100** benchset (final part of Section 3). Ultimately, this work leaves us positioned to recommend a strategy, detailed in the Conclusions, for the development of new benchsets that are designed to embed transferability into ML-DFAs.

**Fig. 1:**
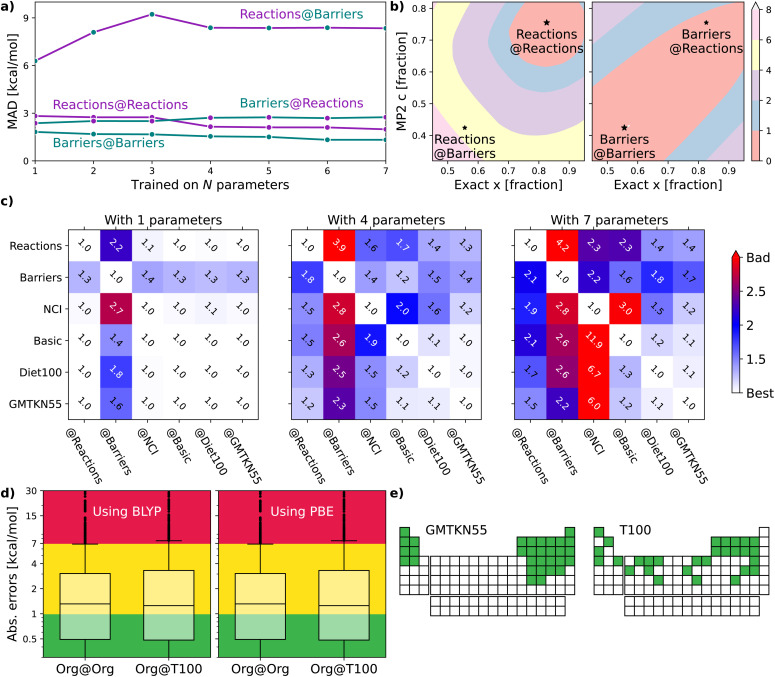
**(a)** Errors for XYG-DFAs with 1–7 parameters applied to subsets covering reaction and barrier chemistry – line colour indicates the test set and dot colour the training set. **(b)** Optimal values for XYG_2_ (2-parameter double hybrid DFA) for **Reactions** and **Barriers** subsets of the GMTKN55 database^16^ (full details of the benchmark sets can be found in Section 5.3). The contours show the MAD in kcal mol^−1^ relative to the optimal value for **Reactions** (left) and **Barriers** (right). **(c)** Transferability matrices between selected benchsets for XYG_1_, XYG_4_ and XYG_7_ (double hybrids with varying parameter number). **(d)** Boxplots with XYG_7_ (one with BLYP and other with PBE semilocal parts) errors for a large organic database (**Org = GMTKN55** excluding **NCI**^16^) with parameters trained on the whole database and on the **T100** benchset (designed from our transferability principles). **(e)** Periodic tables showing the elements (green) included in **GMTKN55** and **T100**.

The following sections will delve into specific details. For now, it suffices to mention that we use a double-hybrid functional form,^18,19^ defined by one^18^ to seven^20^ parameters to controllably train our DFAs. In this way, we generate thousands of data-driven DFAs, to effectively illustrate the utility and analytic power of our TAT. Some key findings of our study are presented in Fig. 1. Fig. 1(a) focuses on our model's efficacy in predicting reaction energies and barrier heights – crucial for calculating thermodynamics and kinetics, respectively.^16^ We train our DFAs on reaction energies and test on barrier heights (**Barriers@Reactions**), and then reverse the sets (**Reactions@Barriers**, full details of the benchmark sets can be found in Section 5.3). From Fig. 1(a) it is clear that our model excels in transferring from reaction energies to barrier heights (thermodynamic to kinetic parameters), but not the other way around. The reason for this asymmetry becomes apparent when we look at the shapes of the cost functions for our two-parameter model and compare the values at their respective minima to those at each other's minima, as shown in Fig. 1(b).

Fig. 1(c) introduces the transferability matrix *T*_**B@A**_, a unitless measure precisely defined as how well a given model trained on arbitrary **A** performs for arbitrary **B** (**B@A**) relative to the accuracy limit of that model for **A@A**. Unlike in Fig. 1(a), which focuses solely on the transferability between reaction energies and barrier heights, Fig. 1(c) includes multiple classes of organic chemical processes.^16^ The matrix provides insights into: (i) transferability for each *T*_**B@A**_ pair; (ii) asymmetry in transferabilities, as shown by differences in *T*_**B@A**_ and *T*_**A@B**_ values; (iii) the rate at which transferability decreases with the increasing number of parameters for different **B@A** pairs; (iv) the chemical classes most transferable to and most transferable from. Transferability matrices are thus a key foundation of our TAT.

Fig. 1(d) demonstrates that two different flavours of our seven-parameter model,^20^ trained on the **T100** benchset (of 100 processes carefully curated around transferability principles of reaction, elemental and transferable diversity), perform as well as their accuracy limits when tested on the extensive 910 process **Org** set, which is the “general-main group thermochemistry, kinetics and noncovalent interactions” (**GMTKN55**) set of 1505 processes, but excluding the 595 non-covalent interactions (**NCI**) to avoid the need for a dispersion correction. ESI Table S1† shows transferabilities between **Org** and **GMTKN55**. This confirms that transferability principles effectively enhance the model's applicability to larger datasets. Fig. 1(e) further highlights the greater elemental diversity in our small **T100** compared to large **GMTKN55**, as it covers a far broader range of groups in the periodic table, despite being fifteen times smaller.

## 2. Transferability assessment tool

To measure transferability from **A** to **B**, we introduce a two-set error MAD_**B@A**_, which is the mean absolute deviation (MAD) on test set **B** for a DFA trained on **A**. We then formulate a unitless transferability matrix:1
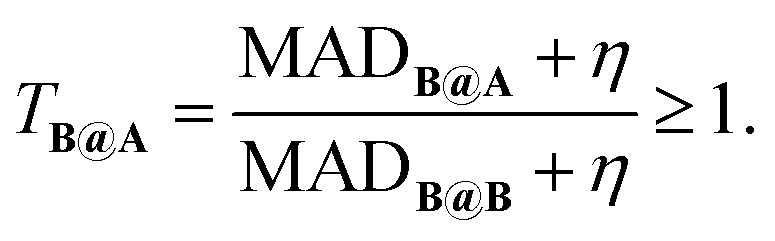
*η* = 0.01 kcal mol^−1^ regularizes results for small energies. By definition, *T*_**B@B**_ = *T*_**A@A**_ = 1 (the case of perfect transferability) and minimization principles dictate that *T*_**B@A**_ ≥ 1, with larger values indicating poorer transferability. Because it involves a ratio, the transferability matrix ensures that errors are normalized by both difficulty, and system size, averaged across the benchset. It thereby complements traditional metrics like MADs.

*T*_**B@A**_ quantifies the performance of a model (DFA) trained on **A** when applied to **B**, normalized by the model's inherent accuracy limit for **B**. Because different kinds of chemistry are sensitive to different kinds of model ingredients, the transferability matrix therefore encodes similarities and differences in the chemistry contained within sets, in a way that is directly relevant to modelling. For example, *T*_**A@B**_ > *T*_**A@C**_ indicates that **C** is ‘closer’ (in terms of chemistry modelled) to **A** than **B** is to **A**. *T*_**B@A**_ > *T*_**A@B**_ indicates that **B** is more sensitive to errors than **A**, and thus A is likely to contain a broader range of chemistry. Finally, *T*_**B@A**_ > *T*_**C@A**_ indicates the chemistry contained in **A** is more useful for **C** than **B**, but not that it is closer.

To use our TAT, we also need to pick a DFA form that can help us to elucidate properties of benchsets. To that end, we use a double hybrid (DH) family of parametrised DFAs, called XYG_*p*_^20^ (named after the original authors^21^), that were designed to systematically switch off empiricism as the number of parameters is decreased, without losing key underlying physics. Here, *p* is the number of empirical parameters varying from one^22^ to seven^20^ (see Methods for the functional forms). We focus on results for one- (XYG_1_), four- (XYG_4_) and seven-parameter (XYG_7_) models to represent minimal, middle and maximal levels of empiricism, but sometimes we explore other numbers of parameters when it is sensible. Along this way we generate hundreds of DFAs for the purpose of analyzing benchsets' transferability.

The DH form is chosen for its generality, as it sits at the top of the current DFA Jacob's ladder (a hierarchy of DFAs based on their mathematical complexity).^23,24^ This allows our DH forms to reduce to functional forms from lower rungs of the ladder during parameter optimization. We use Hartree–Fock (HF) orbitals to calculate all energy terms, to prevent uncontrolled error cancellation of *functional-* and *density-driven* errors when building data-driven DFAs.^22,25^

By varying the level of empiricism, we are able to emulate varying degrees of “machine learning”, without running into issues of genuine machine learning. A typical machine-learned DFA (ML-DFA) may be thought of as an empirical DFA with a very flexible functional form and a very large number of empirical parameters, that are determined by optimising to a training benchset. This flexibility comes at a cost, however, as one (typically) needs to choose:^12,26,27^ (i) the input features, (ii) a neural network (NN) architecture, (iii) a map from NN output to a corresponding DFA, and (iv) benchsets for training, validation and testing. These variables make direct and reproducible tests of transferability tedious and difficult to control. But, by keeping (i–iii) fixed in our case (*i.e.* emulated by a chosen XYG_*p*_ form), and varying (iv) we can focus on the effect of training data in a controlled way. By also varying the number of parameters, *p*, we are able to focus on properties of the benchsets, and not the specific DFA employed, and thus expect any understanding or improvements to benchsets to carry over to ML-DFAs. That is, for present state-of-art deep learned functionals, the XYG_*p*_ model provides a controllable framework that can be used to understand and improve benchsets for uncontrolled fits.

Before concluding this section, we also stress that the transferability matrix concept is not restricted to the MAD, but may be defined for any true metric. For example, Goerigk and Grimme argue^28^ that their WTMAD-2 metric (a weighted average that seeks to equalise weak and strong interactions) is better than MAD for assessing DFAs. By simply replacing MAD by WTMAD-2 in eqn (1) we are able define a TAT for WTMAD-2 that is directly comparable to its MAD counterpart, due to normalisation. Alternately, one might use errors in, *e.g.*, dipole moment in place of relative energies or some other true metric instead of MAD or WTMAD-2. We can even define a transferability matrix between MAD and WTMAD-2 (or any pair of metrics), by evaluating the ratio of WTMAD-2@MAD and WTMAD-2@WTMAD-2 (and *vice versa*), where the “@”’ indicates we optimized XYG_*p*_ using MAD or WTMAD-2. Testing these cross-transferabilities on **GMTKN55** reveals that *T*_WTMAD-2@MAD_ and *T*_MAD@WTMAD-2_ never exceed 1.01 within XYG_*p*_, so MAD and WTMAD-2 are nearly perfectly transferable. We thus consider only MAD for the remainder of this work.

We are now ready to apply the TAT to real data, for the purpose of revealing limitations of existing protocols, and uncovering key principles that enhance transferability and performance across diverse systems.

## 3. Results

Before beginning a detailed analysis of transferability, consider a “minimally-empirical” approach in which a DFA is designed around several fundamental constraints, and then optimised over a small number of processes to determine any remaining parameters. Following Becke's^29^ lead and original XYG_*p*_,^21^ we settle on 3 parameters. The 3-parameter XYG form (*i.e.* XYG_3_) approximately satisfies various constraints by construction.^21^ Training on the 21 ionisation potentials in the benchset **G21IP**^30^ fills in the missing gaps.

At first sight, this seems like an effective strategy: it yields MAD_**GMTKN55@G21IP**_ = 1.91 kcal mol^−1^ across the entire GMTKN55 organic benchset, not far from the optimal MAD_**GMTKN55@GMTKN55**_ = 1.84 kcal mol^−1^ achieved by full optimization of the three XYG_3_ parameters over **GMTKN55**. Using eqn (1), we find a transferability matrix element of 
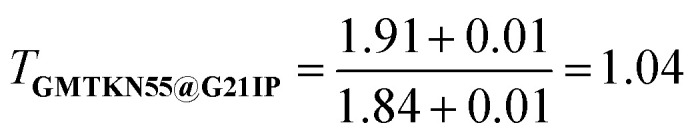
, indicating **G21IP**'s high transferability to **GMTKN55**.

We shall see in the following sections that the construction of **G21IP** that makes it appear as a good candidate for training can be quantified. Deeper analysis, enabled by our TAT, reveals that its success here is an artifact of our choice to use XYG_3_; and that **G21IP** is not a good training set in general. We will show that the TAT enables us to identify and quantify nuances of transferability (or its lack) in different benchsets; and thereby lets us propose three principles that help to embed transferability in training sets. Ultimately, a benchset optimized for transferability will be proposed.

### 3.1 Identifying transferability: concepts learned from organic chemistry

Our goal is motivate transferability principles that can be applied more broadly. As a first step, let us use the key concepts introduced in Section 2 to delve into the details of Fig. 1(a–c), focusing on identifying and understanding transferability within the large **GMTKN55** organic chemistry database.

Fig. 1(a) shows that training barrier heights (194 processes^16^) on reaction energies (243 processes^16^) performs nearly as well as training on barriers themselves. However, reaction energies perform poorly when trained on barriers, suggesting either barriers are easier to learn or that reactions are better for training. Fig. 1(b) explains this result and lets us pick the right conclusion for the case of a two-parameter XYG_2_ (the parameters being exact exchange fraction and MP2 correlation fractions). Errors in **Barriers** are rather insensitive to changes in parameters, meaning that picking sub-optimal paramet ers does not lead to major additional errors. Not so for errors in **Reactions**, where curvature is much sharper and, consequently, changing parameters rapidly worsens results. Therefore **Barriers** are easier to learn than **Reactions**.

The *T*_**B@A**_ transferability matrices in Fig. 1(c) for XYG_1_, XYG_4_, and XYG_7_ show how transferability rapidly worsens as the number of model parameters increases, characteristic of over-fitting. In the 1-parameter case, many *T*_**B@A**_ values are close to 1.0, indicating high transferability. Conversely, in the 7-parameter model, numerous entries exceed 3, implying performance three times worse than optimal. The upper 4 × 4 block highlights transferabilities among four test subsets: **Reactions**, **Barriers**, **NCI**, and **Basic**^16^ (everything else, such as atomization energies, ionization potentials, proton/electron affinities, *etc.*). The block reveals that **Reactions** is the most transferable training set, indicated by the smallest values in its column. Conversely, **Basic** appears to be the most challenging to transfer to, as evidenced by the largest values in its row. In the ESI,† we show *T*_**B@A**_ by further breaking down GMTKN55's subsets (ESI Fig. S7–S9†). Interestingly, within XYG_1_, reaction sets are more transferable to barriers than different barrier sets are to each other (ESI Fig. S7†).

Furthermore, Fig. 1(c), with *T*_**B@A**_ for multiples sets (see ESI Fig. S12† for the corresponding MAD_**B@A**_ figures), already challenges the obvious, and so far dominant in data-driven DFA development, strategy of increasing the size of datasets. **Diet100** (with 100 processes) does a much better job as a training set than any of the larger (∼250 processes) ‘chemistry’ subsets; and performs nearly as well as **GMTKN55** at predicting **Reactions**, **Barriers** and **Basic**. Unfortunately, the way **Diet100** was constructed offers no useful insights for improving transferability principles, although it does convincingly confirm that quality is more important than quantity.

Fortunately, **GMTKN55** comprises 55 subsets (34 of which are in **Org**), each representing (more-or-less) different types of chemistry and enabling numerous transferability analyses. *e.g.*, we observe strong transferability of reaction energies between smaller and larger molecules (see ESI Fig. S11†), and we can measure the transferability between relative energies of charged *versus* neutral species (see ESI Fig. S10†). Furthermore, we can leverage GMTKN55's diversity to develop a better understanding of transferability and use it to create the T100 set, explicitly engineered for high transferability, as hinted at in Fig. 1(d) and (e). We will revisit the last two panels of Fig. 1 after elaborating on the essential principles that inform this set's design.

### 3.2 Transferability principle 1: reduce human bias in the training set to embed genuine reaction diversity

Consider a hypothetical experiment involving two distinct groups: chemistry students and art students. Given a molecular editor and specific drawing rules (*e.g.*, use no more than 16 spheres in total and stick to the colors white, gray, blue, *etc.*), the optimized structures and benchmarked energies from their drawings would form the basis for two different empirical density functionals (‘Art’ and ‘Chemistry’ functionals). We will show that functionals trained on the art students' molecules would easily outperform those based on the chemistry students' designs. The latter group's chemical intuition is to blame, as it introduces unexpected biases in the data.

**Fig. 2:**
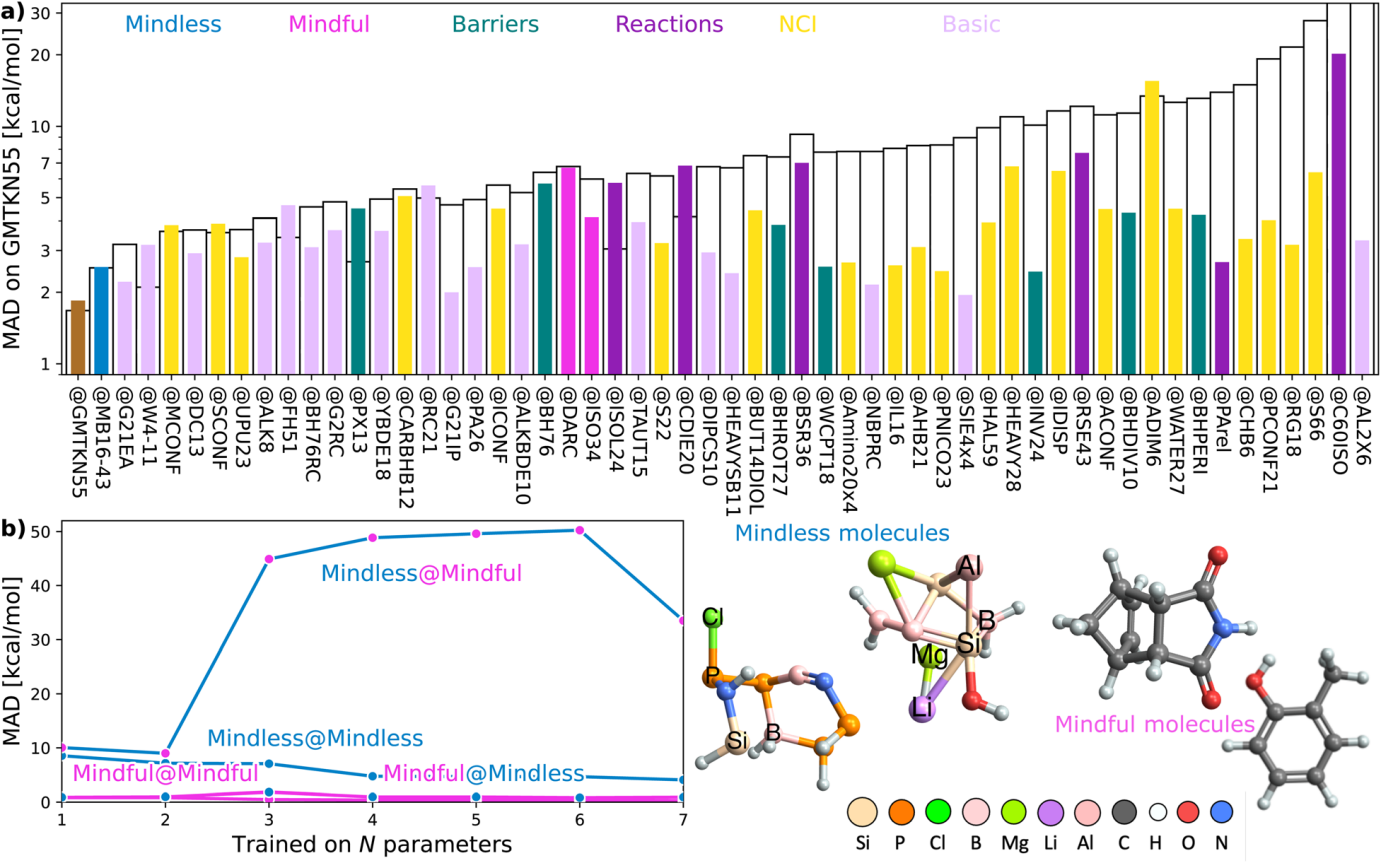
**(a)** Mean absolute deviation (MAD, log scale) for **GMTKN55@subset**, where subset is a **subset** of GMTKN55. The order reflects the MAD and absolute difference between XYG_3_ and XYG_7_. **(b)** Errors for DFAs with 1–7 parameters applied to subsets covering mindless and mindful construction of benchmark set. Some example mindless and mindful molecules are shown at right.

To begin, let us play a game where we optimize our DFA models for each of the 55 subsets within **GMTKN55** and then assess how well each of the 55 resulting DFAs transfers to the full **GMTKN55** database. Fig. 2(a) shows the key results from this game, displaying MADs for **GMTKN55@subset** from each of the 55 subsets, using 3- and 7-parameter models, XYG_3_ (as employed in our example using **G21IP**) and XYG_7_ (the most empirical DFA in the XYG family). In most cases, MAD for XYG_3_ and XYG_7_ are vastly different, and even when they are not, MAD are very large. These indicators of poor transferability reflect the fact that different subsets capture different chemistry and do not represent the whole GMTKN55 in this specific transferability context.

Returning to our opening example, we see that **G21IP** performs well with XYG_3_ but poorly with XYG_7_ – its transferability is strongly influenced by the number of free parameters (ESI Fig. S2† further highlights this point when both XYG_3_ and XYG_7_ are applied to non-covalent interactions). In the case of XYG_3_, **G21IP** was able to discriminate a good functional space from a bad (*i.e.* poorly transferable) one, but that was not the case when the number of parameters increased to 7. Indeed, **G21IP** is not unique in that regard – transferability for XYG_7_ is almost always worse than XYG_3_. Increasing parameters elevates the risk of overfitting challenging us to identify datasets whose transferability remains robust despite additional parameters. While regularization strategies applied to a DFA form (through *e.g.*, physical constraints) can enhance its transferability,^31,32^ our TAT has a different focus that complements this regularization strategy. Namely, eqn (1) allows us to see how transferability varies with different training sets for any optimizeable DFA form, enabling us to identify general principles for the design of training sets with improved transferability.

Transferability principle 1 is revealed by the standout performer in Fig. 2(a): MB16-43,^33^ which yields low errors with just 43 data points (W4-11 has 140). What is special about **MB16-43**? It is the only subset in GMTKN55 that is not biased toward chemical intuition or the limited chemical space it spans. Simply put, unlike the remaining 54 subsets, its structures have not been manually drawn by humans before undergoing geometry optimizations. Rather, MB16-43 avoids unnoticed human bias *via* “mindless” (more accurately, a clever random strategy) construction of molecules – we shall henceforth denote it as **Mindless** to stress this feature.

Fig. 2(b) shows that DFAs trained on **Mindless** (43 processes) predict good energies for a similarly-sized more **Mindful** (DARC + ISO34 with 48 processes covering Diels–Alder and isomerisation reaction energies^16^) selection of data. But, the reverse is not true – **Mindless@Mindful** has up to six-fold increases in errors compared to **Mindless@Mindless**. Our results thus confirm that mindless benchmarking achieves its goal of “[making] use of random elements constrained by systematic and controllable specifications to avoid unsystematic and uncontrolled criteria”.^33^ The small size of **Mindless** again stresses the importance of quality over quantity.

Furthermore, the transferability captured by **Mindless** is independent of both the **Mindful** dataset (ESI Fig. S15†) and the semilocal part of our functional (ESI Fig. S16†). We therefore see that **Mindless** captures genuine diversity of chemical interactions – *i.e.*, it achieves transferability principle 1. In simpler terms, **Mindless** (art students) molecules yield far better functionals here than **Mindful** (chemistry students) ones.

### 3.3 Transferability principle 2: span the periodic table to embed elemental diversity

Modern technologies rely on most of (stable) elements in the periodic table.^36^ By contrast, two thirds of processes in GMTKN55 contain only C, H, N, O or F. This highlights a second limitation of the training data we have considered so far – a lack of elemental diversity. Improving elemental diversity is the most intuitive of the transferability principles, yet we shall see it still throws up some surprises.

Before beginning our analysis, it is worth highlighting some recent work^17^ that shows how vitally important diversity in training benchsets can be. Zhao *et al.*^17^ revealed that DM21 (trained on organic chemistry sets and some exact limits) cannot even converge to a self-consistent solution in multiple transition metal systems, including atoms. The difficulty of extrapolating from organic chemistry to TMs is intuitive to anyone familiar with DFA development, although such a dramatic failure of DM21 is still surprising. On the other hand, our TAT matrices show that transferability rapidly decreases with the number of parameters, making the catastrophically poor extrapolation of DM21, with its roughly half a million parameters, more foreseeable. Nevertheless, the question remains: how can we avoid such catastrophes?

GMTKN55 completely excludes transition metals [Fig. 1(e) shows the elements of the periodic table that GMTKN55 covers], so we turn to TMC151,^34^ a 151-process benchset based around transition metal (TM) chemistry, to introduce some inorganic chemistry into our game and supplement the results of GMTKN55. Despite the sparsity of TM benchmarking (151 *versus* 1505 processes) we are nonetheless able to develop an understanding of transferability between main group and TM chemistry by using the TAT to explore relationships between (subsets of) TMC151 and GMTKN55.

**Fig. 3:**
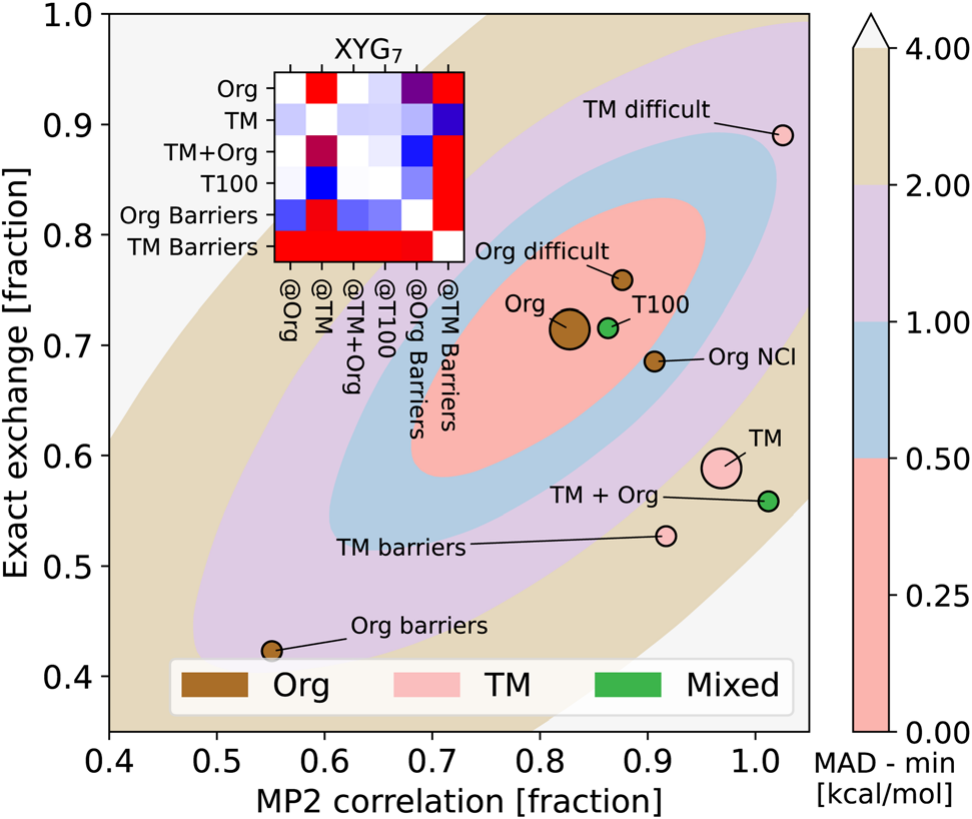
Optimal values for the two-parameter model (markers) for organic (**Org**) and transition metal (**TM** = TMC151 (ref. 34)) processes, and subsets thereof (*e.g.*, TMOR = metal–organic reactions^34,35^). Also shows the MAD (contours) of organic processes as a function of the two parameters, zeroed at the minimum. Inset: XYG_6_ transferability matrix for selected Org and TM sets.

Fig. 3 reveals that training on main group elements is not a good strategy for predicting transition metal chemistry, or *vice versa*, even in the simple XYG_2_ model (chosen because it can be visualised). The optimal parameters for TM sets live in a different region of the parameter space compared to those for the main group sets. Transferability from TMC151 (denoted **TM** to stress its focus on transition metals) to **Org** (*i.e.* GMTKN55 excluding NCIs) is very poor, as can be seen from the contour plots (for XYG_2_) and inset transferability matrix (for XYG_7_). Simply adding the two sets (**TM + Org**) improves results in general, but still has transferability issues for both **Org Barriers** and **TM Barriers** (see inset). Note, while the optimal parameter space in Fig. 3 may seem surprising at first, the differences between the optimal spaces of standard double hybrids and those applied to Hartree–Fock orbitals, which we use here, are often notable.^22^ Having the MP2 correlation fraction over 1 in Fig. 3 is neither unexpected nor an issue, given that MP2 is generally not exact and that its errors can cancel that of DFA.

In view of the extremely poor transferability of DFAs trained on TMs to Org, adding elemental diversity (*e.g.*, molecules with 3d elements) to a main-group training set could ruin the good accuracy of DFAs for Org (further highlighted in ESI Fig. S23†). However, as we shall soon see, this risk is completely eliminated once the training set is diversified in a manner that explicitly favors transferability. Thus, what we seek in a training set is not just elemental diversity, as this can come with drawbacks. Instead, what we want in the training set and what we advocate for is a balance between genuine reaction diversity, elemental diversity and transferable (chemical) diversity – to be defined soon. **Mindless** gave us our first hint that human intuition may be counterproductive to such a goal. We will now proceed to show how it can be achieved more systematically.

### 3.4 Transferability principle 3: embed transferable diversity to maximize transferability

After adding some TM into the game, we are ready to return to the last two panels of Fig. 1, where we showed some results for our new benchset, **T100**. The most important feature of **T100** is that it is explicitly designed around three transferability principles: (1) it randomly selects chemical processes from **TMC151+GMTKN55** to yield genuine reaction diversity; (2) it includes a bias in construction toward genuine elemental diversity; (3) it is optimized to improve average transferability in the XYG_1_, XYG_4_ and XYG_7_ functional forms, giving us a final ML-DFA that is explicitly designed to give good transferability. The principles behind the first two have already been discussed. Full details are in Methods and ESI Sec. S2.†

Importantly, the third design feature for **T100** provides an implicit definition of transferable diversity: a benchset has transferable diversity if an approach trained on it is transferable to (*i.e.* performs well on) other benchsets. Despite being (or because it is!) the least intuitive of the three transferability principles, transferable diversity is the most important principle. It tells us that simply increasing the number of processes or elements in a benchset is not enough to improve its usefulness as a training set. We need to make sure that what we add will improve training. Put more explicitly, transferable diversity is the property that “chemistries” are appropriately weighted or proportioned in the benchset, so as to improve predictions without accidental bias. For example, Mindless clearly has good transferable diversity, despite having significantly less elemental diversity than **T100**.

The boxplots in Fig. 1(d) indicate that XYG_7_ trained solely on the 100 chemical processes in **T100** performs nearly as well as when trained on all 910 **Org** processes. This holds for both the BLYP-based XYG_7_ model used in **T100** creation; and a PBE-based XYG_7_ variant that has not been seen during the construction of **T100**. The differences between the two are described in Methods. Fig. 1(e) shows that T100 covers a far broader range of periodic table groups than **GMTKN55**, despite the two containing similar numbers of elements. Fig. 1(d and e) thus reveal the effectiveness of embedding transferability principles into data curation.

**Fig. 4:**
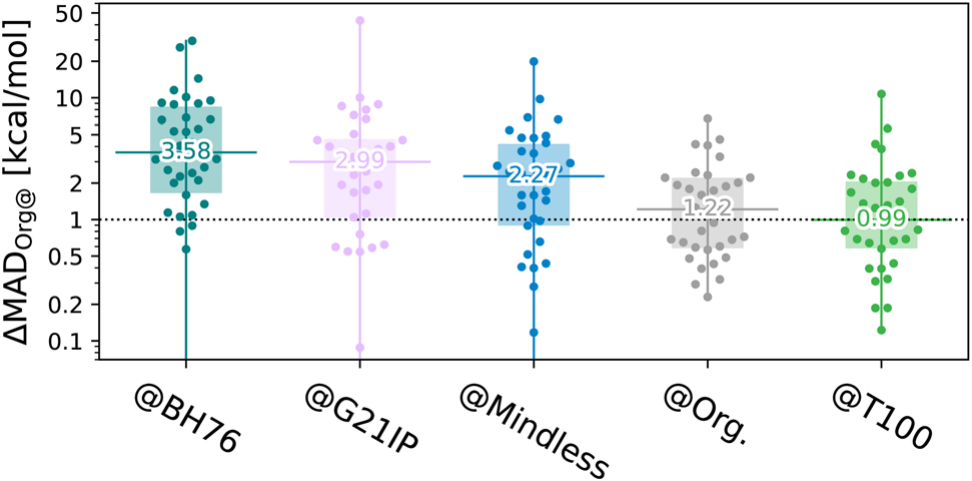
Transferability energy (log scale – note, some outliers are below the plot) of the 34 subsets of **Org** trained on different benchsets, for a 7-parameter XYG-DFA. Beeswarm plots^37^ show the 34 benchsets, horizontal lines and numbers indicate the median, boxes indicate the 1st–3rd quartiles.

The results shown in Fig. 4 highlight that the T100 optimisation strategy has very useful consequences for the transferability energy cost,

2 ΔMAD_**B@A**_ = MAD_**B@A**_ − MAD_**B@B**_ ≥ 0.

ΔMAD_**B@A**_ yields the difference in energy between actual and optimal performance when a DFA is transferred from a training set to a test set and thus supplements *T*_**B@A**_ by quantifying the energy cost of using the ‘wrong’ instead of optimal parameters. In Fig. 4, **B** is any of the 34 subsets of **Org** while **A** (listed below each figure) is the training benchset, used to optimise XYG_7_. We see that both **BH76** and our old friend **G21IP** provide poor training data, leading to excess errors of over 1 kcal mol^−1^ in 75% of subsets. Thus, the poor results of Fig. 2(a) are not caused by a small number of outliers, but are systematic.

By contrast, **T100** actually outperforms **GMTKN55** when applied to diverse organic chemistry, albeit as a consequence of our choice to sample by set. This is despite being optimized to balance transferability between main group and TM chemistry [remember the periodic tables for the two sets shown in Fig. 1(e)]. Indeed, 70% of benchsets are predicted to within 2 kcal mol^−1^ of their optimal (self-trained) values. Nonetheless, T100, as a sample of GMTKN55 and TMC151 designed for enhancing transferability in training, cannot be compared to the extensive GMTKN55 database for method testing.

Table 1 reports results for 7-parameter DFAs tested on a diverse list of example benchsets; and reveals that, 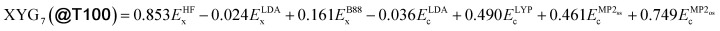, introduces only modest errors compared to a very high target – the best possible result for each set (@Self, that is MAD_**B@B**_). Interestingly, this DFA has more exact exchange and MP2 correlation than other double hybrids,^18,21,38^ in part because we use HF orbitals as inputs.^22^ High amounts of exact exchange and MP2 correlation also enable XYG**@T100** to give high accuracy for self-interaction-error (SIE) related problems which are typically challenging even for double hybrids^22^ (see Fig. S24 and S25† for further examples for the related SIE4 × 4 set). However, XYG_7_**@T100** is less accurate for transition metal barriers (TMB), yielding four times larger MAD than XYG_7_**@TMB**. Going back to Fig. 4, training on mindless benchmarks (**@Mindless**) is a little worse on average, but still better than using **@Mindful** molecules. Results for r^2^SCAN (with different optimal parameters) follow a similar trend.

**Table 1:** MAD (kcal mol^−1^) for different datasets (rows) of the XYG_7_ functional trained on the datasets given in columns. Results shown for BLYP- and r^2^SCAN-based XYG_7_

Set	@Self	@T100	@Mindless	@Mindful
**BLYP**				
S66	0.18	0.34	0.33	0.32
W4-11	2.58	4.58	6.85	57.38
Water27	0.08	0.82	4.82	6.08
BH76	1.41	3.70	3.11	4.96
OrgDiff	5.41	7.59	8.87	37.24
ISOL24	0.36	1.36	1.65	0.86
TMB	1.21	4.83	5.75	4.37

**r** ^**2**^ **SCAN**				
S66	0.21	0.41	0.36	0.71
W4-11	2.41	3.46	4.43	32.25
Water27	0.06	1.36	0.98	5.35
BH76	1.77	3.13	3.10	4.77
OrgDiff	6.11	7.89	7.70	18.06
ISOL24	0.51	2.17	1.52	0.94
TMB	1.85	5.06	5.50	5.65

### 3.5 The accuracy limit and focus on difficult cases

Finally, the TAT also lets us evaluate the accuracy limit of double hybrids – that is the **A@A** case, which is the best possible results for a specific kind of problem given the double hybrid functional form. We remind the reader that XYG_7_(**A**) is optimized over all seven parameters, so represents the best possible pure (*i.e.* not range-separated) double hybrid for a given benchset **A**. Therefore, MAD_**A@A**_ indicates the smallest possible error from our XYG_7_ double hybrid family and dictates its accuracy limit.

**Fig. 5:**
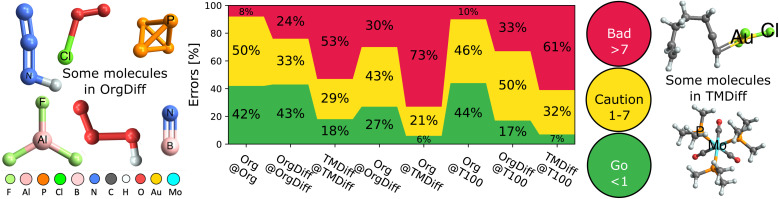
Fraction of processes with good (<1 kcal mol^−1^), ok (1–7 kcal mol^−1^) and bad (>7 kcal mol^−1^) errors, MAD_B@A_. Includes selected optimal (**B** = **A**) and suboptimal (**B** ≠ **A**) combinations. Some example difficult molecules are illustrated to the left (**Org**) and right (**TM**).

Fig. 5 explores the accuracy limits of double hybrid functional forms by showing the distribution of absolute errors for various benchsets, with a focus on difficult cases.^34,39^ It reports a selection of optimal (self-optimized **A@A** cases) and non-optimal (**A@B** cases) DFAs, to reveal that the overwhelming majority of organic processes can be predicted with good (<1 kcal mol^−1^; chemical) or ok (1–7 kcal mol^−1^; useful) accuracy, so long as they are trained on a good reference benchset (here, **Org** or **T100**).

But, Fig. 5 also reveals that difficult cases, particularly in transition metals, remain elusive. A quarter (24%) of difficult organic (**OrgDiff**)^39^ and half (53%) of difficult transition metal (**TMDiff**)^34^ processes exceed acceptable error margins, even with the optimal DFAs. Supp. Fig. S26† reveals that errors cannot be explained by spin-contamination or low-quality benchmarks. Despite generally excellent performance on main group chemistry, current DFA strategies are simply not ready to address true chemical diversity (mechanism and elements) with standard functional types even when using ingredients from all rungs of Jacob's ladder.^23,24^

Moreover, DFAs trained on these difficult cases perform poorly on the full **Org**, especially compared to the almost “best case scenario” of **T100** as a training set. Furthermore, this poor performance is reciprocal – using **T100** as a training set for **OrgDiff** or **TMDiff** also significantly worsens prediction.

There is a plus side, however, as difficult cases for DFAs are often also difficult cases for the (very expensive) creation of benchmarking data. The accuracy limit suggests that benchmark quality (and thus cost) may therefore carefully be relaxed in some difficult cases.

## 4. Discussion and conclusions

This work provides an alternative conceptual framework for identifying and understanding chemical diversity, as it pertains to model transferability. Central to our results is the transferability assessment tool (TAT), and the scenario where one dataset serves as a training set and another as a test set, and then their roles are reversed. This (indeed simple) consideration, encoded in the TAT matrix, uncovers critical insights into the suitability of various training sets, shifting the paradigm from intuition-based to rigorously evidence-based methodology in empirical electronic structure method development. The TAT, in tandem with the XYG_*p*_ protocol, provides a wealth of analytic information about the training and testing of data-driven DFAs. We can use it to identify what chemistry is hard to learn, what kinds of processes are useful to train on, and to answer many of the questions posed in the introduction.

The main conclusion from our work is that following transferability principles to embed transferability in data curation is crucial for the construction of general-purpose models in chemistry. By following these principles, a training benchset should embed genuine chemical and elemental diversity; in such proportions within the benchset that they improve transferability (*i.e.* with good transferable diversity). The evidence presented here therefore suggests the following strategy for better construction, optimization and refinement of benchsets that can be used to train complex, data-driven DFAs:

**(1)** Human input/bias should be reduced in the creation of training (and test) sets, in favour of randomness in chemical construction;

**(2)** Elemental diversity of training sets should be improved, possibly *via* lower quality benchmarks;

**(3)** Training sets and DFAs should be optimized and refined with an explicit bias toward improving transferability, by testing transferability matrices during their construction.

Our work has revealed that both Mindless (=**MB16-43**, Fig. 2 and 4) and **T100** (Fig. 1, 3–5) make large steps in the right direction: Mindless eschews pre-determined chemistry and **T100** embeds diversity and transferability, both by design. The mindless strategy can be (i) adapted to other cases (*e.g.*, mindless ionization potential or barrier height benchsets); (ii) further extended by introducing randomness in the selection of mindless potential energy surface points, which are not confined to local minima; (iii) biased toward elemental and transferable diversity [as done for **T100**, eqn (5) below] to construct entirely new benchsets. Furthermore, we envision that using TAT within active learning frameworks can aid in directing learning towards the most significant regions of chemical space for use in training. In practice, this could be achieved by using the TAT to choose which datapoints go into the training – an active learning extension to the creation of our **T100**.

The catastrophic failure of DM21 for some TMs^17^ clearly highlights why embedding transferability at the training benchset stage is vitally important. By contrast, the success of **Mindless** and **T100** as training sets for diverse chemistry highlights how we can potentially do much better with more careful selection of training data. Embedding transferable diversity by using the transferability principles therefore becomes imperative for machine-learned DFAs. Otherwise, better interpolation on chemistry seen in training risks poorer extrapolation to (prediction of) chemistry unseen in training.

It is also worth stressing that the TAT may be applied to embed transferability into any empirical model, and especially those for which the level of empiricism can be controlled. This includes models based on wave function theories (at one extreme) and machine learning of ‘classical’ energies from molecular geometries (at the other extreme). Work along these lines should be pursued.

Finally, it is important to note that transferability principles are important to consider even for models that explicitly target a specific type of chemistry problem (*e.g.* DFAs optimized for organic barriers or materials chemistry). Despite their narrower goals, such approaches implicitly assume that the training benchset contains sufficient diversity to enable predictions of similar problems; and that the diversity is appropriately weighted. The low transferability between subsets of **Barriers** reveals that these assumptions are not guaranteed. Embedding transferable diversity into training benchsets, even for narrowly-focussed problems, enables higher confidence in their predictive reliability.

## 5. Methods

### 5.1 XYG DFAs

All XYG_*p*_ functionals considered in this work have the same fundamental functional form,3
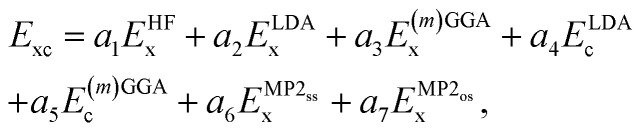
where *E*_x(c)_ indicate exchange (correlation) energy approximations, *E*^HF^_x_ is the exact HF exchange energy and 
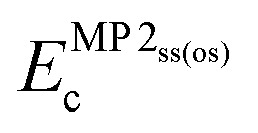
 indicate the same-spin and opposite-spin parts of the MP2 energy. *E*^(*m*)GGA^_x_ and *E*^(*m*)GGA^_c_ denote GGA or *meta*-GGA exchange and correlation.

The DFA of eqn (3) is thus defined by a seven-component vector, 
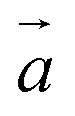
. XYG_7_ allows flexible choice of all seven components. For XYG_*p*<7_, the components of the vector are determined by the following rules:

*p* = 1: choose exact exchange fraction, *α*, and set *a*_1_: = *α*, *a*_2_: = *a*_4_: = 0, *a*_3_: = 1 − *α*, *a*_5_: = 1 − *α*^2^, *a*_6_: = *a*_7_: = *α*^2^

*p* = 2: choose exact exchange fraction, *α*, and MP2 fraction, *β*, and set *a*_1_: = *α*, *a*_2_: = *a*_4_: = 0, *a*_3_: = 1 − *α*, *a*_5_: = 1 − *β*, *a*_6_: = *a*_7_: = *β*;

*p* = 3: choose free *a*_1_, *a*_3_ and *a*_6_, and set *a*_2_: = *a*_4_: = 0, *a*_5_: = 1 − *a*_6_, *a*_7_: = *a*_6_;

*p* = 4: choose free *a*_1_, *a*_2_, *a*_3_ and *a*_6_, and set *a*_4_: = 0, *a*_5_: = 1 − *a*_6_, *a*_7_: = *a*_6_;

*p* = 5: choose all except *a*_4_: = 0 and *a*_7_: = *a*_6_;

*p* = 6: choose all except *a*_7_: = *a*_6_.

Unless otherwise specified, throughout this work we use Becke's (B88)^40^ exchange GGA and Lee, Yang and Parr's (LYP)^41^ correlation GGA for *E*^(*m*)GGA^_x_ and *E*^(*m*)GGA^_c_, respectively (BLYP). The optimal DFA for set A is then defined *via*,4
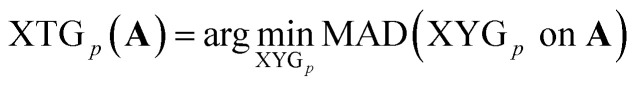
where XYG_*p*_ indicates all possible variants of eqn (3) consistent with the number, *p*, of parameters (using BLYP as GGAs); and MAD(DFA on **set**) indicates the mean absolute deviation of energies computed using DFA, averaged across all processes in set. We thereby obtain, MAD_**B@A**_: = MAD(XYG_*p*_(**A**) on **B**).

The results for two other combinations—PBE exchange + PBE correlation;^42^ and r^2^SCAN exchange + r^2^SCAN correlation^43^—are given in the ESI.† The main conclusions of our work do not change once we replace the BLYP-based GGAs with their PBE-/r^2^SCAN-based counterparts in eqn (3).

### 5.2 Computational details

All HF and DFT calculations were conducted with Orca 5.0.0.^44^ We used def2-QZVPPD for GMTKN55 and def2-QZVP for TMC151. For costly cases, def2-QZVP(P) or def2-TZVP(P) were used. Further details, including the description of our robust minimizer for obtaining the XYG_*p*_ parameters, are in Sec. S1 of the ESI.† Orbitals were computed using unrestricted Hartree–Fock (UHF) theory in all cases.

### 5.3 Special benchmark sets

Mostly we use the categories from GMTKN55 and TMC151 or preexisting subsets (*e.g.***Diet100** (ref. 45)). We also have some special benchset (and aliases to stress important features):

**Mindless** is an alias for MB16-43,^16,33^ to stress its most important feature;

**Mindful** combines DARC and ISO34 sets;^16^ chosen to represent chemical intuition-based counterpart of Mindless;

**Org** indicates GMTKN55 with the non-covalent interaction (NCI) subsets excluded, to focus on typical organic chemistry;

**Org difficult = OrgDiff** is the P30-5 ‘poison’ subset of GMTKN55, from ref. 39;

**Org X** indicates a subset from GMTKN55;

**TM** is an alias for TMC151, to stress its focus on transition metal chemistry;

**TM difficult = TMDiff** is a subset of TMC151 composed of TMD + two MOR41 reactions + six TMB barriers, all identified as difficult in ref. 34;

**TM X** indicates a subset from TMC151;

**TM + Org** is the combination of Org and TMC151;

**T100** is a subset of **TMC151 + GMKTN55** designed to embed transferable diversity principles.

Interestingly, there is a perfect transferability between Org. and the NCI subset of GMTKN55 - *T*_**B@A**_ for this pair never exceeds 1.01 for the used XYG models. For further descriptions of the used (sub)sets, please see Table S2† in the ESI.†

#### 5.3.1 T100 construction

To construct **T100** we first ‘mindlessly’ breed twenty “pretty transferable” (denoted **PT**_1…20_) subsets of the combined GMTKN55 and TMC151 (**TM + Org**) benchset, each with 100 processes. Survival is dictated by a genetic approach similar to that used to construct Diet sets, with breeding success based on transferability of XYG_7_.^45^ Full details are in Section S2 of the ESI.† Then, we obtain **T100** by selecting the best one, using:5
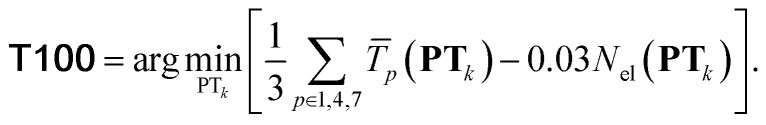
Here, 
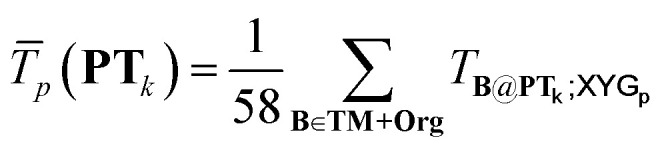
 is the average transferability from PT_*k*_ to all 58 subsets of GMTKN55 and TMC151, using XYG_*p*_. Averaging over *p* ∈ 1, 4, 7 helps to avoid ‘accidental’ transferability for any specific number of parameters. Biasing to a larger number, *N*_el_(**PT**_***k***_), of unique elements in **PT**_***k***_ helps to avoid over-representation of main group chemistry, which is 10 times more common than TM chemistry in **TM + Org**.

We use BLYP (Becke exchange^40^ and Lee–Yang–Parr correlation^41^) in eqn (3) for both the breeding and optimisation stages, which means the transferable diversity of T100 is biased toward BLYP. In principle, other functional choices might lead to other sets. Nevertheless, ESI Fig. S27† reveal that training PBE- and r^2^SCAN-based XYG_*p*_ on BLYP's **T100** gives them transferability similar to DFAs trained on the full GMTKN55 benchset. **T100** also works for a different functional form – that of B3LYP,^29^ which excludes MP2 contributions entirely (see ESI Fig. S28†). It follows that transferable diversity features of **T100** are largely independent of functional form choice.

## Code availability

The code is provided on the GitHub repository https://github.com/vuckovic-lab/transferability for this work (see “read.ipynb” notebook for explanations on how to generate the data from the code).

## Data availability

ESI† supporting this article is available. Additional data can be generated using the code described in the code availability statement.

## Author contributions

SV conceived the transferability presented here and carried out most computations. TG and SV worked together on analysis (including coding) and writing. SD helped with chemical insights. BC helped with insights into benchmarking and computation. All authors contributed to editing and review.

## Conflicts of interest

There are no conflicts to declare.

## Supplementary Material

SC-015-D4SC02358G-s001
