# Supplementary material for: Identifying and embedding transferability in data-driven representations of chemical space
Source: Chem Sci. 2024 Jun 21;15(28):11122–33. doi: 10.1039/d4sc02358g (PMC11253166; doi:10.1039/d4sc02358g)
Supplement: SC-015-D4SC02358G-s001 [file SC-015-D4SC02358G-s001.pdf]

Electronic Supplementary Material (ESI) for Chemical Science.
This journal is © The Royal Society of Chemistry 2024

Supplementary material for "Identifying and embedding transferability in data-driven representations of chemical space"

Tim Gould,[1] Bun Chan,[2] Stephen G. Dale,[1,3] and Stefan Vuckovic[4]

[1] *Queensland Micro- and Nanotechnology Centre, Griffith University, Nathan, Qld 4111, Australia*

[2] *Graduate School of Engineering, Nagasaki University, Bunkyo 1-14, Nagasaki 852-8521, Japan*

[3] *Institute of Functional Intelligent Materials, National University of Singapore, 4 Science Drive 2, Singapore 117544*

[4] *Department of Chemistry, University of Fribourg, Fribourg, Switzerland.*[a]

[a] Electronic mail: stefan.vuckovic@unifr.ch

CONTENTS

S1. Computational details S2

S2. Breeding of "pretty transferable" benchsets S4

S3. Transferability between GMTKN55 and Org S6

S4. Transferability of functionals trained on G21IP set S7

S5. Further details on the results in Figure 1(a) S8

S6. Further details on the results in Figure 1(c) S9

S7. Further details on the results in Figure 2(a) S18

S8. Details on the Mindful vs. Mindless analysis S20

S9. Further details on the results in Figure 3 S22

S10. Additional Details for TM vs Organic chemistry transferability S28

S11. Additional results for SIE4x4 set S30

S12. Additional results for the accuracy of @T100-based functionals S32

S13. Datasets description S33

References S34

S1. COMPUTATIONAL DETAILS

All unrestricted HF/DFT calculations have been performed from the Orca 5.0.0 package within the def2-QZVPPD basis set for GMTKN55 subsets and def2-QZVP for TMC151 subset. For a small number of reactions when this basis was too expensive, we settled for:

- def2-QZVPP - for the ISOL24 and C60ISO sets.
- def2-TZVPP - for the UPU23 set.

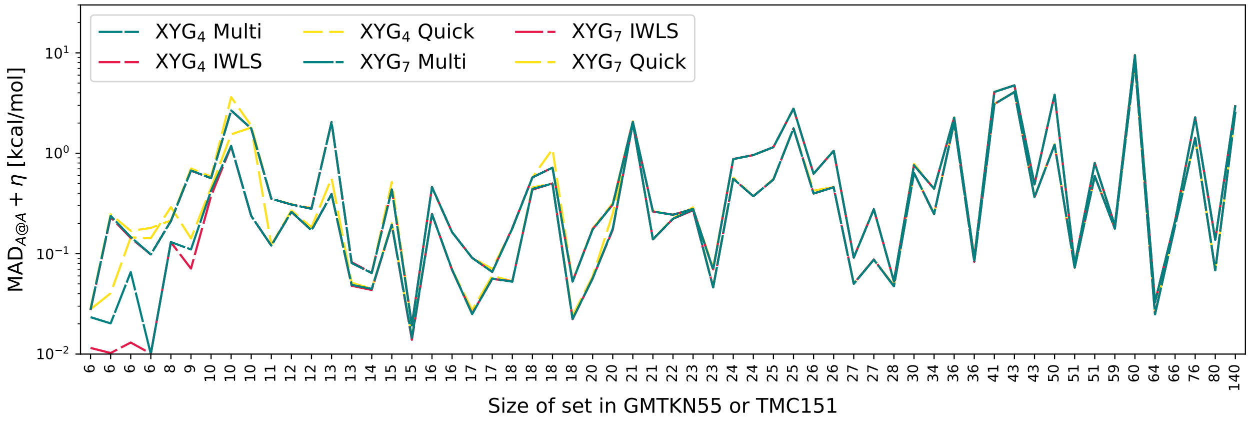

FIG. S1: Optimal MAD for all subsets of GMTKN55 and TMC151 computed using the three optimization strategies, shown as a function of set size. Results are shown for XYG$_4$ and XYG$_7$ models, with the number of parameters indicated by subscripts in the legend.

- def2-TZVP - for the two MOR reactions involving "ed24", "ed25", "pcy3", "pr24", "pr25" molecules

Furthermore, we have used *RIJCOSX* for approximating Coulomb and HF Exchange in our calculations and "TightSCF" Orca keyword for tight SCF convergences. For larger elements, when appropriate, we have used: def2-ECP effective core potentials (associated with the def2- basis set family).

For the optimization of for each DFA considered, we employ three strategies to optimize the MAD as a function of input parameters:

1. **Multi**: Scipy's *scipy.optimize.minimize* function was utilized, employing the default BFGS algorithm in tandem with our *multiple-seed strategy*. This strategy uses the optimization from various initial seeds, incorporating both a set of statically defined seeds and dynamically generated pseudo-random seeds. For the static seeds, each parameter in $\{a_i, \ldots, a_N\}$ is set to one of the values in $\{0.125, 0.25, 0.75, 1.0\}$. Additionally, M dynamically pseudo-random generated seeds are used, with the default $M = 10$ found to be sufficient for the robustness of the optimization process.

2. **IWLS**: Iterative re-Weighted Least Squares (IWLS) is used to optimize the MAD. This involves iterating a weighted least squares problem: $\mathbf{c}^{(i)} = (\mathbf{X}^T\mathbf{W}^{(i-1)}\mathbf{X} + 10^{-5}\mathbf{I})^{-1}\mathbf{X}^T\mathbf{W}^{(i-1)}\mathbf{Y}$ where $\mathbf{X}$ and $\mathbf{Y}$ are the usual matrices used for least squares minimizaion of, $\sum_k |y_k - \mathbf{X}_k\mathbf{c}|^2 = \mathrm{tr}[(\mathbf{Y} - \mathbf{X}\mathbf{c})^T(\mathbf{Y} - \mathbf{X}\mathbf{c})]$; and $\mathbf{W}^{(i)}$ is a digaonal

matrix with elements $1/\max(10^{-4}, |y_k - \mathbf{X}_k \mathbf{c}^{(i)}|)$, i.e. one over the absolute error. The numbers 10^{-5} and 10^{-4} are regularisation parameters to aid in convergence.

3. **Quick**: A crude least squares fit is first used to minimize the RMSD as a guess for the MAD, and then *scipy.optimize.minimize* is used to refine the guess. This approach is only used during the training of T100.

The effectiveness of our strategies can be seen in practice. Theoretically, $T_{i@j}$ should never be less than zero. The occurrence of $T_{i@j} < 1$ would therefore suggest that the optimization of a a DFA trained on dataset "j" is not optimal and that a lower minimum can be found. However, in practice, instances of $T_{ij} < 1$ were never observed (even for large matrices), evidencing the robustness of our multiple-seed optimization strategy in identifying optimal DFA parameters. As further evidence, Figure S1 shows that **Multi** and **IWLS** are indistinguishable in performance for datasets with more than 10 elements (covering all considered in detail in this work) so may be used interchangeably. For larger sets, **Quick** (only used to optimize **T100**) is also indistinguishable from the more robust but slower approaches.

S2. BREEDING OF "PRETTY TRANSFERABLE" BENCHSETS

In the main text, we formulate and motivate [see eq. (1) and eq. (last) and surrounding discussion] the unitless transferability of **A** to **B** and mean transferabilities of set **A**:

$$T_{\mathbf{B@A};p} = \frac{\mathrm{MAD}_{\mathbf{B@A};p} + \eta}{\mathrm{MAD}_{\mathbf{B@B};p} + \eta}\,, \qquad \bar{T}_p(\mathbf{A}) = \frac{1}{58} \sum_{\mathbf{B} \in \mathbf{TM+Org.}} T_{\mathbf{B@A};p} \tag{S1}$$

T measures the error on set **B** when optimized on **A** (**B@A**) versus its minimum error when optimized on itself (**B@B**). $\eta = 0.01$ kcal/mol regularizes results for small energies. $\bar{T}$ is the mean of this error over *all* 58 subsets of **TM+Org.**. Here, p indicates the number of parameters used in the optimization.

A random set involves 100 processes selected at random out of the 1656 processes of **GMTKN55+TMC151**, and we create $N_{\text{initial}} = 1000$ of these. $\bar{T}_7(\mathbf{rand}_R)$ will serve as our metric for breeding the pretty transferable sets, $\mathbf{PT}_K$ from $\mathbf{rand}_R$ – i.e. we use transferability on XYG$_7$. We therefore also define $C_7 := N_{\text{initial}}^{-1} \sum_{R=1}^{N_{\text{initial}}} \bar{T}_7(\mathbf{rand}_R)$ to be the mean transferability obtained by chance, which we use as a normalizing factor.

Our goal is to construct 20 pretty transferable sets, via the following algorithm:

1. From the random sets, $\{\mathbf{rand}_R\}$, select the sets with the $N_{\text{survive}} = 100$ lowest values of $\bar{T}_7$, to form a breeding pool, $\{\mathbf{breed}_B\}$.

2. Breed $\{\mathbf{breed}_B\}$ to create a single pretty transferable set:

   (a) Select the best [smallest $\bar{T}_7(\mathbf{breed}_B)$] set, $\mathbf{breed}_\alpha$, from the breeding pool, and another set, $\mathbf{breed}_\beta$, at random from the rest of the breeding pool;

   (b) Breed a new set, $\mathbf{breed}_\gamma$, that contains all N_S processes shared by $\mathbf{breed}_\alpha$ and $\mathbf{breed}_\beta$, and fills the remaining $100 - N_S$ processes by random selection from unshared elements of $\mathbf{breed}_\alpha$ and $\mathbf{breed}_\beta$;

   (c) Replace the worst [largest $\bar{T}_7(\mathbf{breed}_B)$] set in the breeding pool by $\mathbf{breed}_\gamma$, or leave the list unchanged if $\bar{T}_7(\mathbf{breed}_\gamma)$ is higher;

   (d) Repeat from step 2a for up to $N_{\text{breed}} = 2000$ times, or until all $\bar{T}_7(\mathbf{breed}_B)$ are within $0.001C_7$ of each other.

3. Define $\mathbf{PT}_K = \mathbf{breed}_\alpha$, set $K \to K + 1$ and repeat from step 1 (resetting the breeding pool each time) until 20 sets have been created.

The code `BreedTransferable.py` implements the above algorithm, while the code `PickBest.py` implements the second stage of T100's construction that is described in the main text. The pool of pretty transferable sets used to select T100 is cached (see `PickBest.py` for location) and read by `PickBest.py` to allow for reproducibility. All files are provided on the `github` repository [`https://github.com/vuckovic-lab/transferability/`] for this work (see "read.ipynb" notebook for pedagogical explanations on how to extract the data from the code).

S3. TRANSFERABILITY BETWEEN GMTKN55 AND ORG

TABLE S1: Transferabilities between **GMTKN55** and **Org** (i.e. GMTKN55 with non-covalent interactions removed) for XYG_p with p from one to seven.

	XYG_1	XYG_2	XYG_3	XYG_4	XYG_5	XYG_6	XYG_7
$T_{\mathbf{Org@GMTKN55}}$	1.00	1.00	1.00	1.00	1.00	1.01	1.01
$T_{\mathbf{GMTKN55@Org}}$	1.00	1.00	1.00	1.00	1.00	1.01	1.01

S4. TRANSFERABILITY OF FUNCTIONALS TRAINED ON G21IP SET

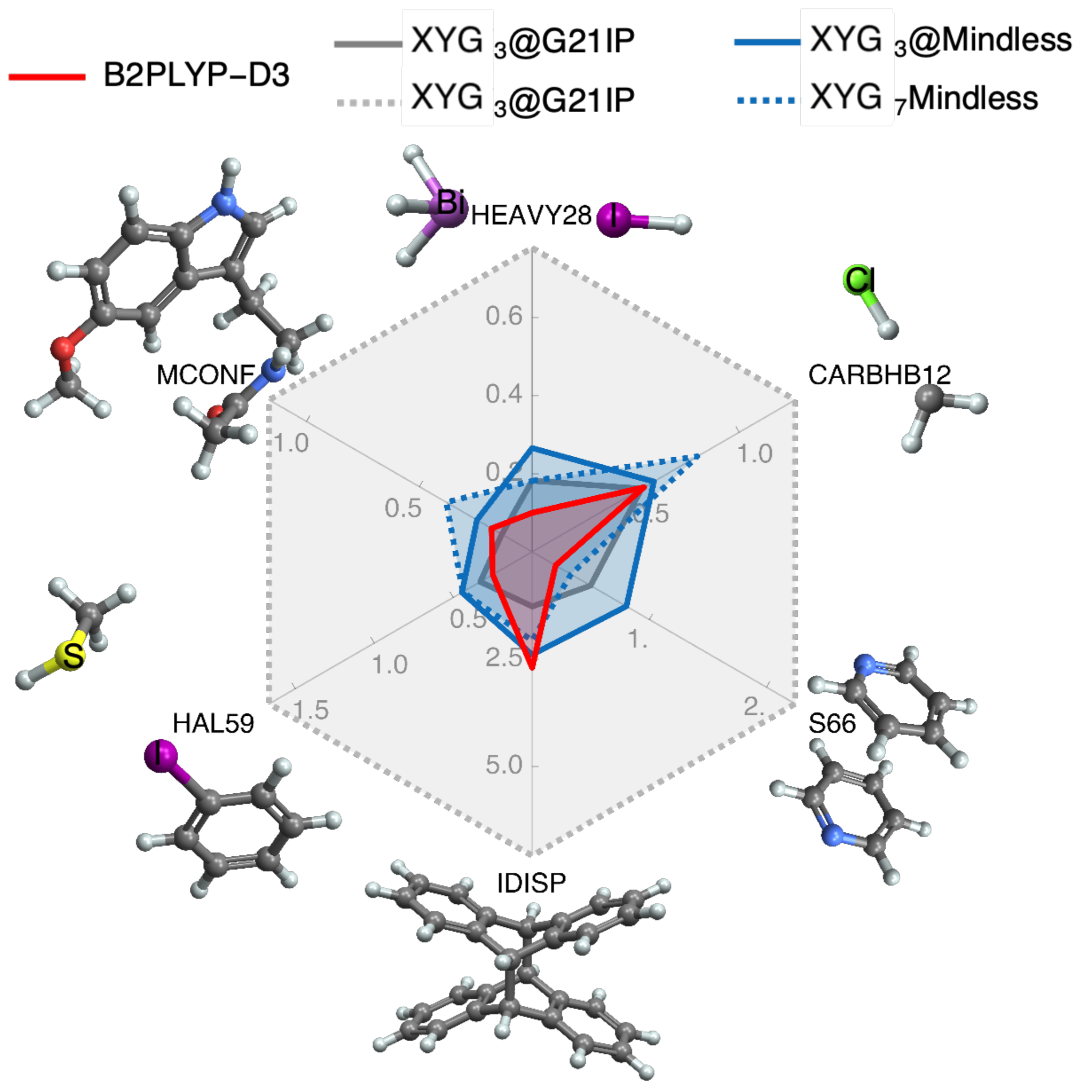

FIG. S2: Radial plots displaying MAD [kcal/mol] of selected functionals across different NCI subsets within the GMTKN55 database. The XYG_3@G21IP functional, trained only on 21 ionization potentials, performs on par with or better than B2PLYP-D3, despite the former having no D3 dispersion correction. However, this transferability diminishes when extending from 3 to 7 parameters. Conversely, XYG_N@Mindless functionals maintain consistent NCI performance as N transitions from 3 to 5.

S5. FURTHER DETAILS ON THE RESULTS IN FIGURE 1(A)

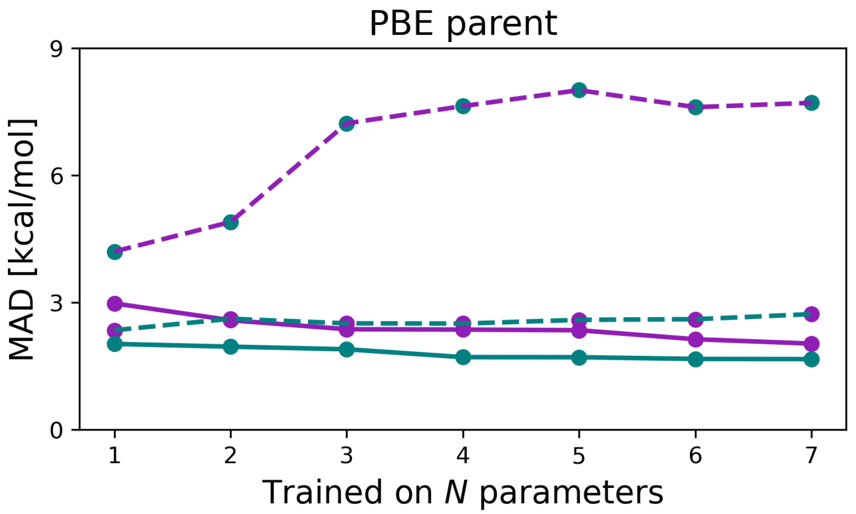

(a) PBE parent

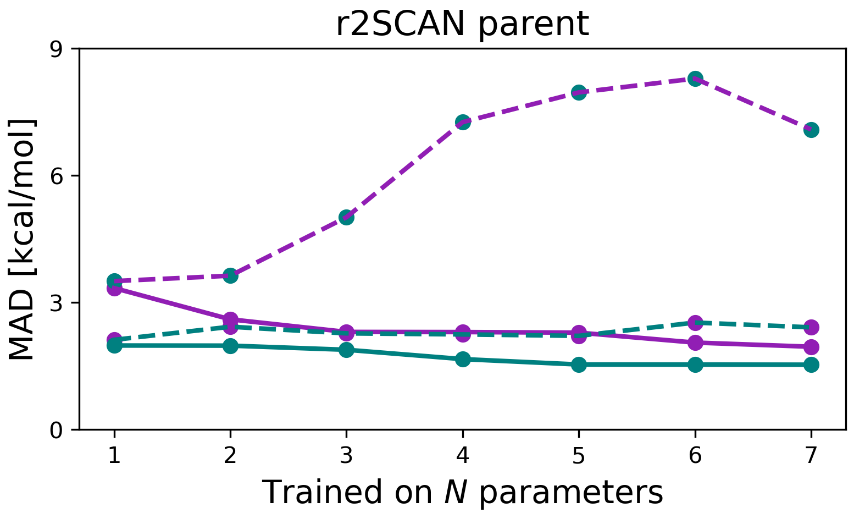

(b) r^{2}SCAN parents

FIG. S3: Same as figure 1(a), but with PBE and r^{2}SCAN parents, purple represents reactions energies (R), while teal color represents barriers (B): purple line with purple beads represents R@R, purple line with teal beads represents R@B, teal line with purple beads represents B@R, teal line with teal beads represents B@R.

S6. FURTHER DETAILS ON THE RESULTS IN FIGURE 1(C)

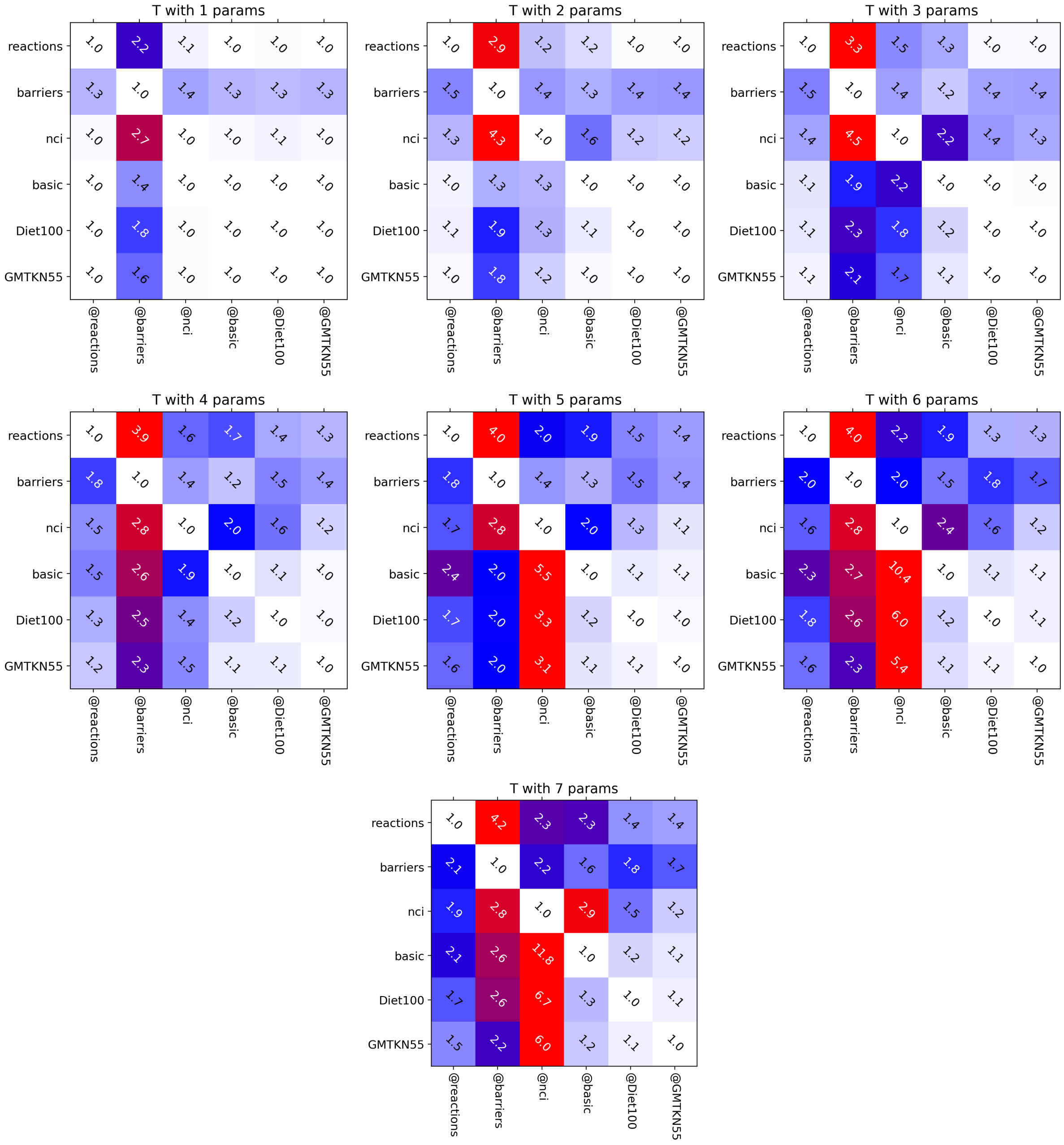

FIG. S4: Same as Figure 1(c), but for all number of parameters between 1 and 7.

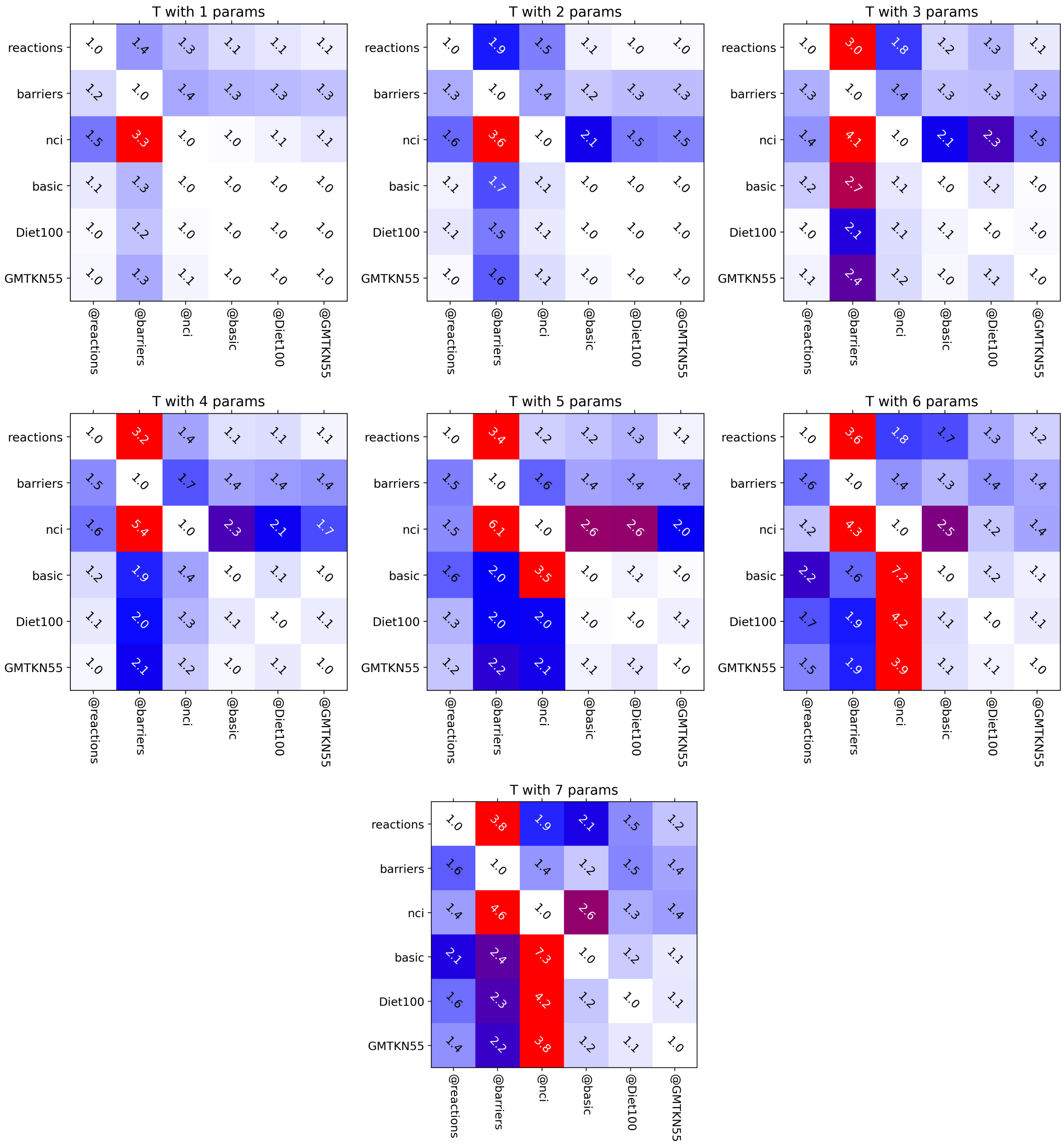

FIG. S5: Same as Figure S4, but for PBE parent.

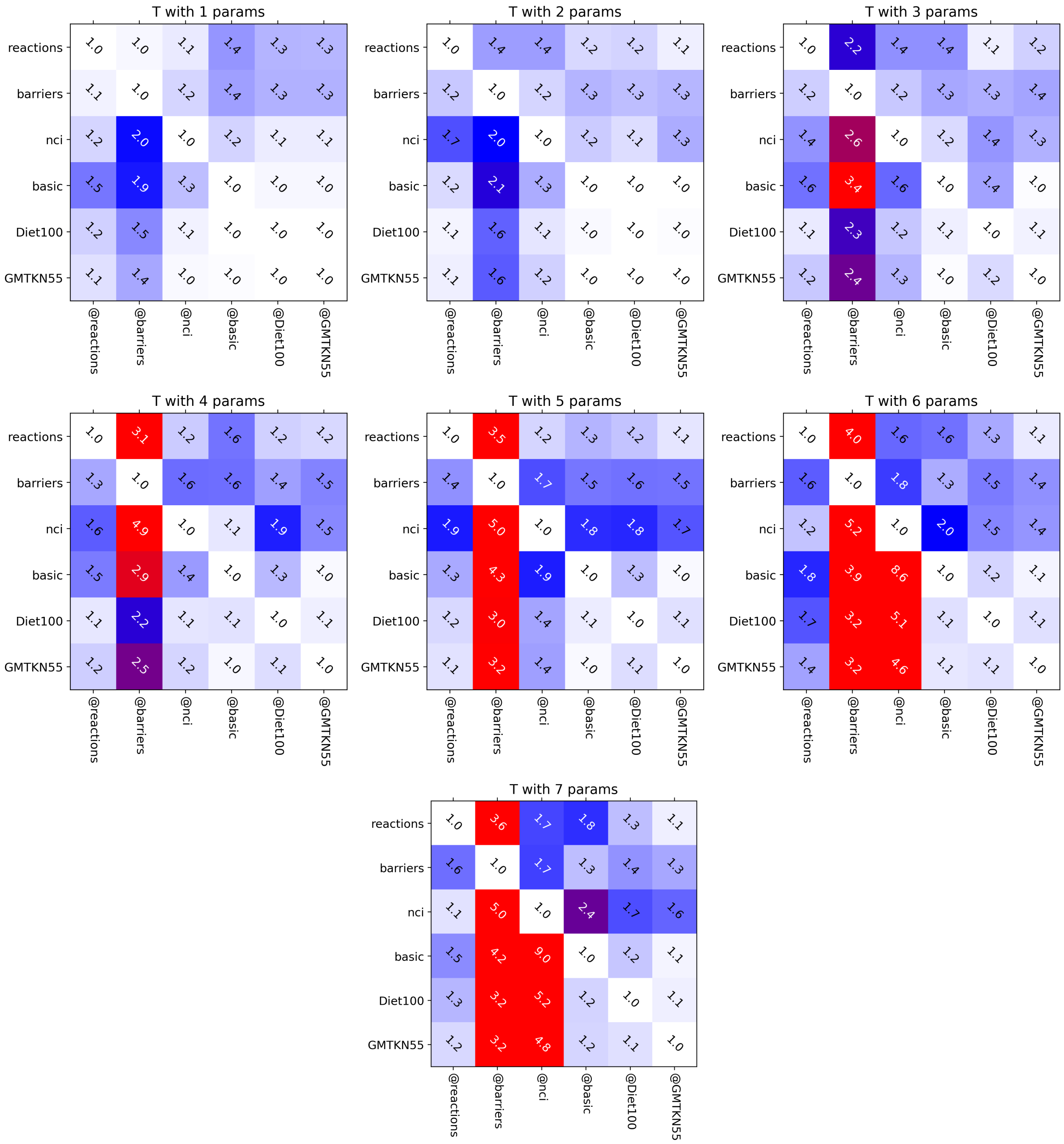

FIG. S6: Same as Figure S4, but for r^{2}SCAN parent.

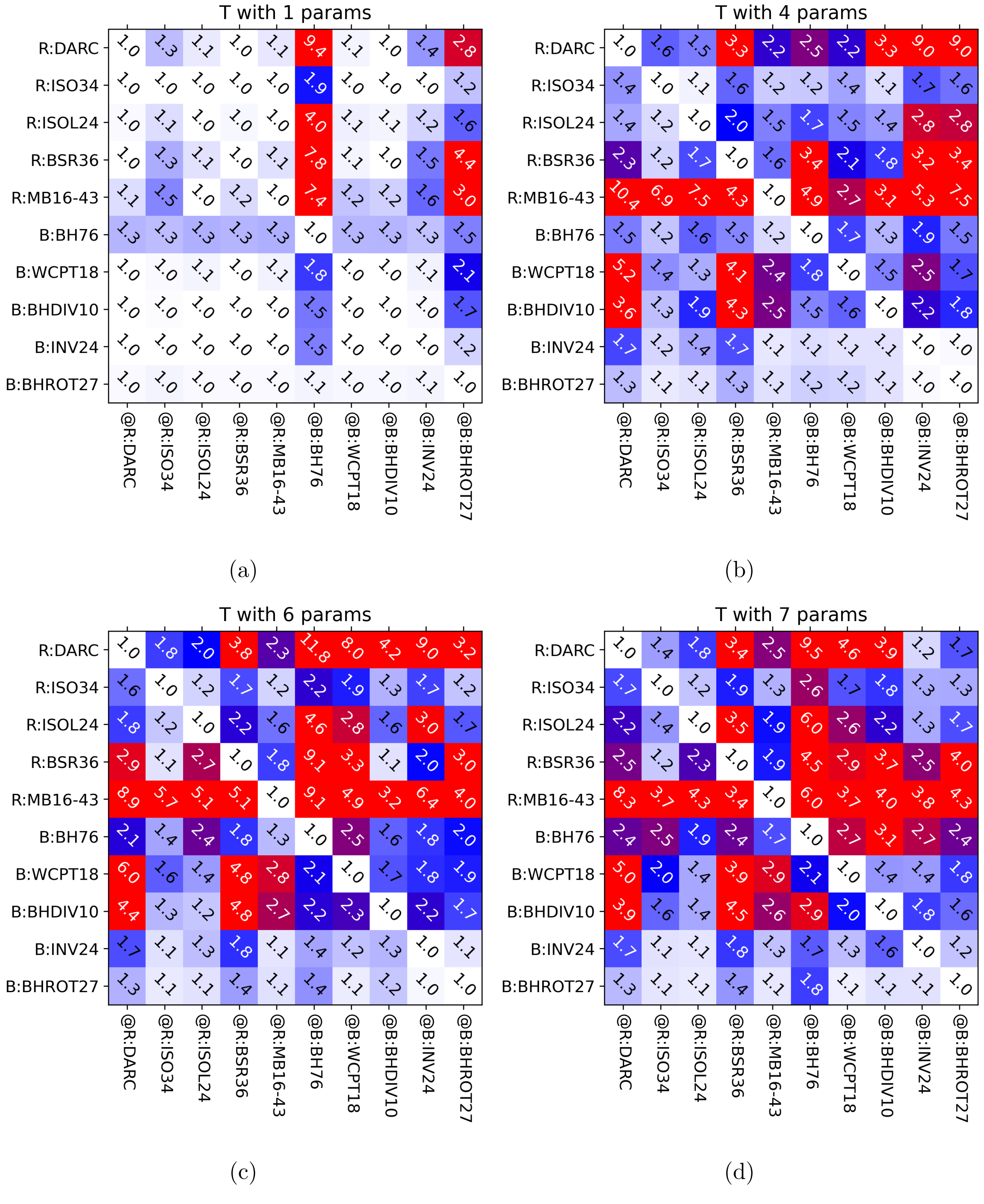

FIG. S7: Transferability matrices for reaction and barrier subsets of GMTKN55. **R@R** and **B@B** blocks of the matrices show the intra-reactions and intra-barriers transferability. **R@B** blocks show how barriers transfer to reactions. **B@R** blocks show how reactions transfer to barriers. η set to 1kcal/mol as the denominator of the transferability matrix for a single GMTKN55 subset becomes very small.

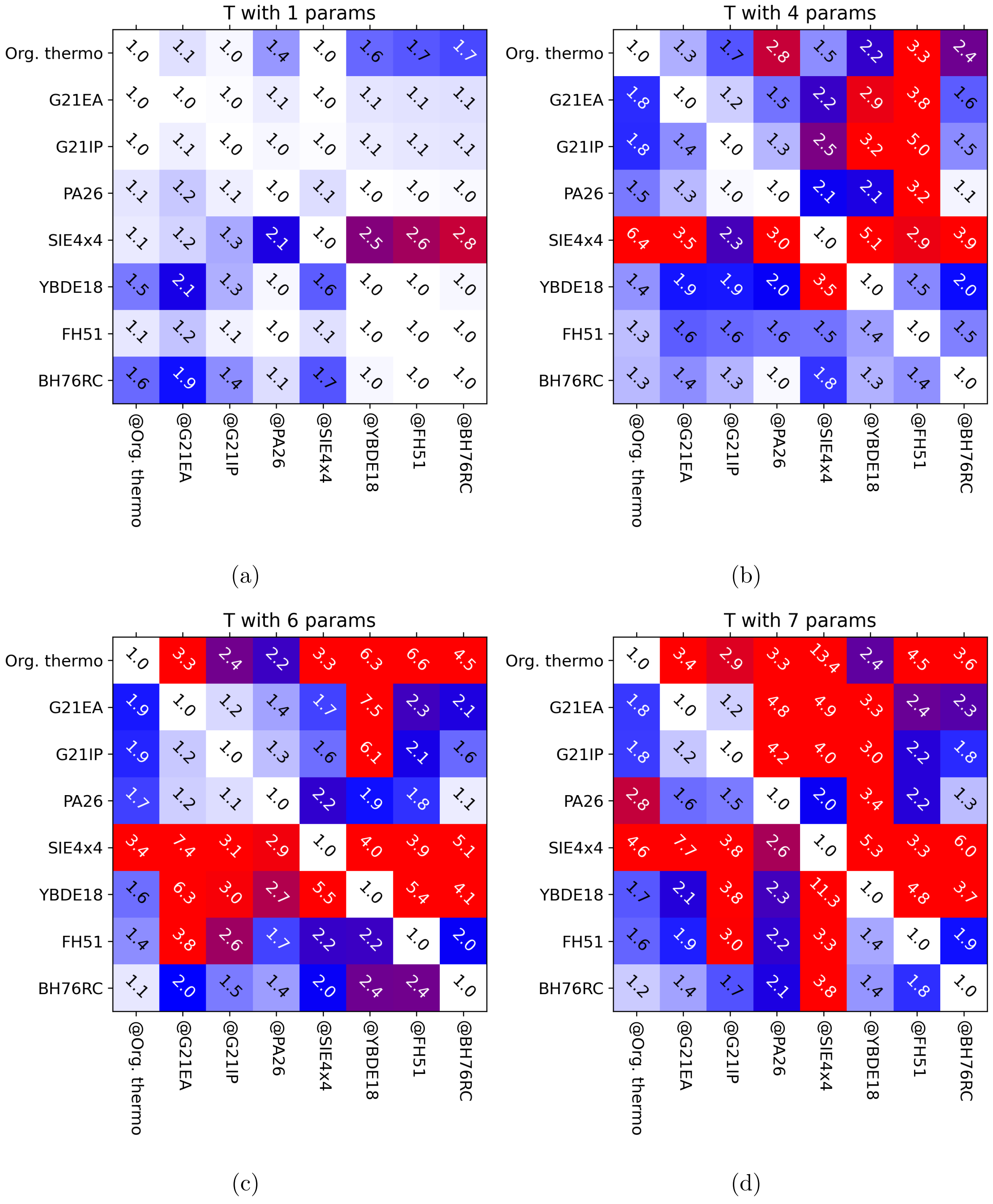

FIG. S8: intra-"Basic" Transferability matrices for the sets belonging to the "Basic" part of GMTKN55. η set to 1kcal/mol as the denominator when train only a single subset of GMTKN55 can get very small. "Org. Thermo" represents "W4-11" set.

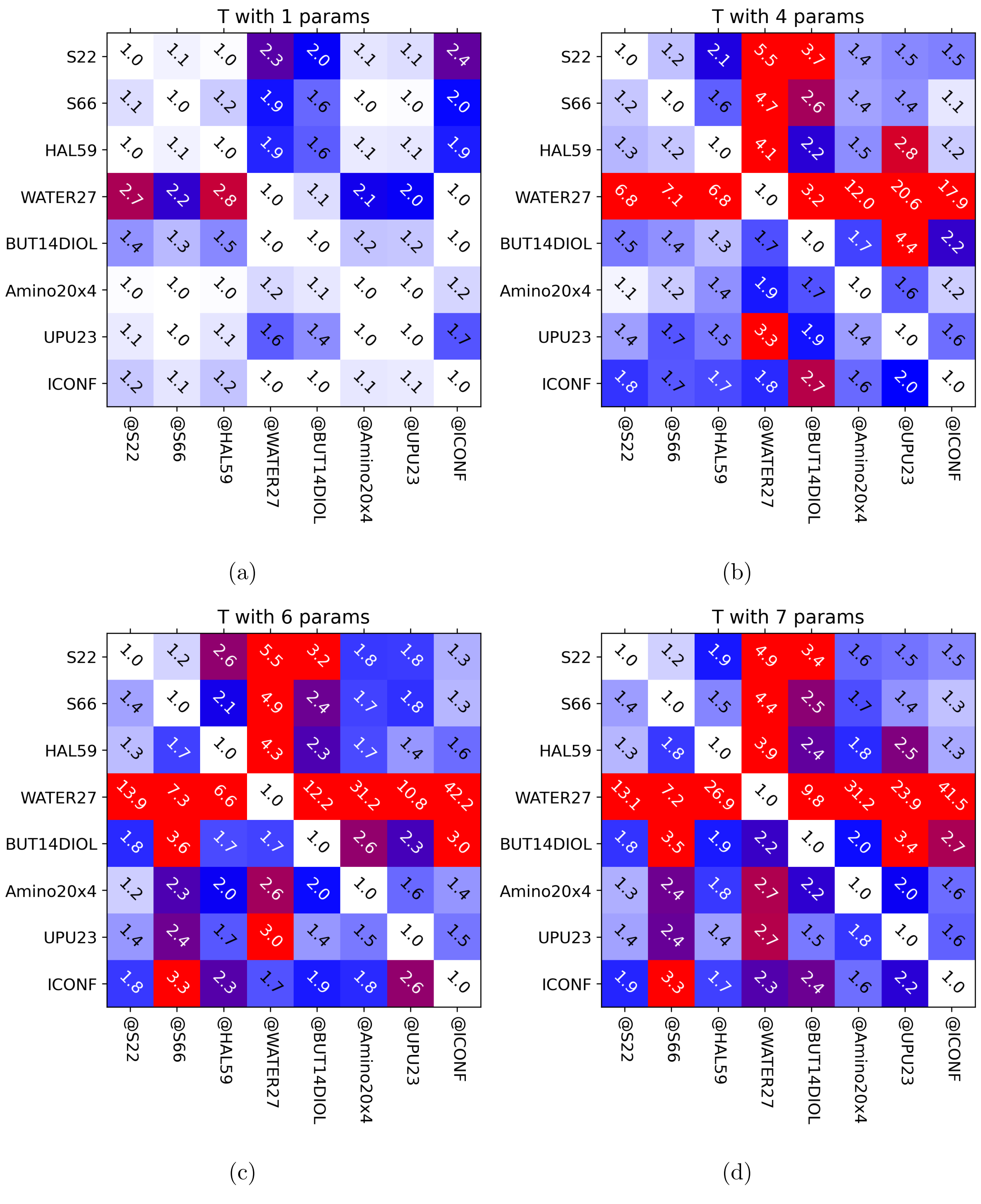

FIG. S9: Transferability matrices for the sets belonging to the "inter-NCI" part [first 4 sets] and "intra-NCI" [last 4 sets] of GMTKN55. η set to 0.1 kcal/mol.

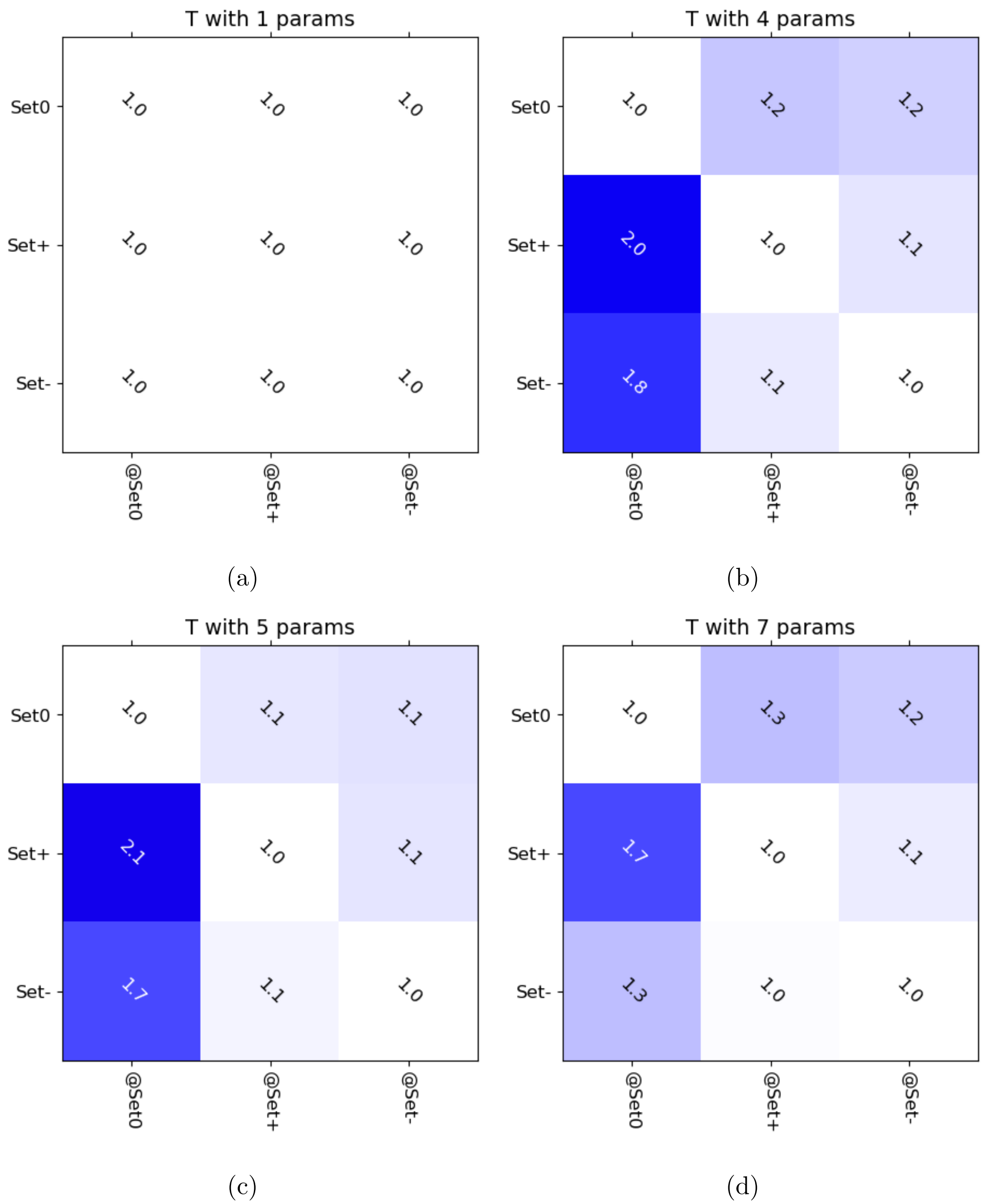

FIG. S10: Transferability matrices for the "Set0", "Set+", "Set-" sets with varying number of parameters within XYGp double hybrid which uses BLYP as a GGA [same as Fig. 1(c)]. η is set to 0.5 kcal/mol. Set0 includes atomization energies of 45 randomly chosen neutral molecules from W4-11. Set+ includes atomization energies of 20 randomly chosen neutral molecules from W4-11 and 25 (randomly chosen) ionization potentials from the G21IP dataset. Set- includes atomization energies of 20 randomly chosen neutral molecules from W4-11 and 25 electron affinities from the G21EA dataset (whole G21EA set).

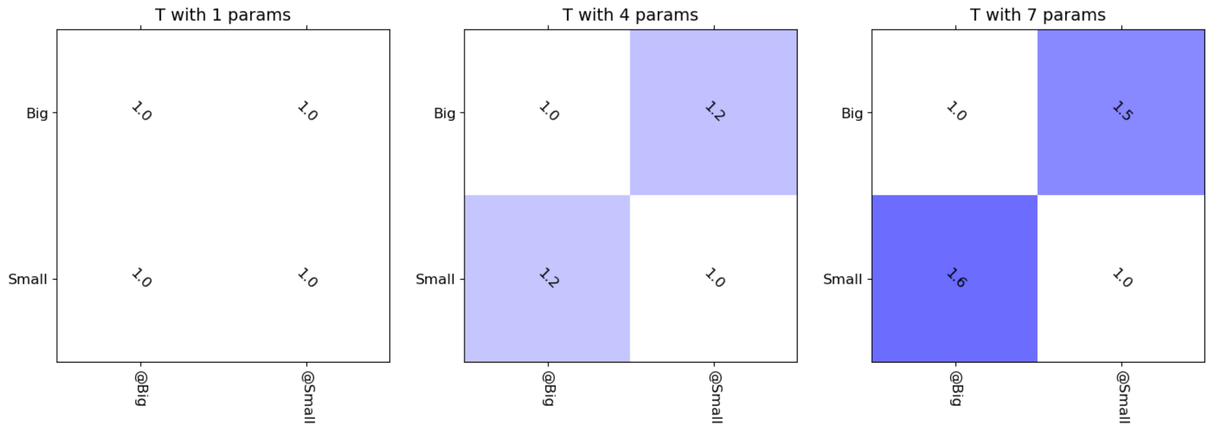

(a) (b) (c)

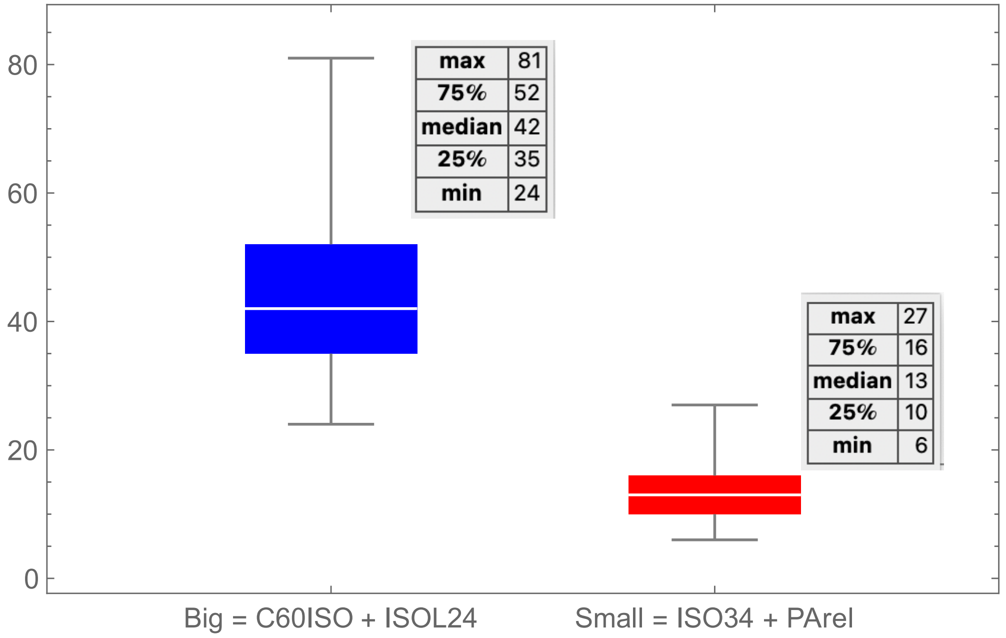

(d)

FIG. S11: (a)-(c): Transferability matrices for the "Big", "Small" sets with varying number of parameters within XYGp double hybrid which uses BLYP as a GGA [same as Fig. 1(c)]. η is set to 0.5 kcal/mol. "Big" includes C60ISO and ISOL24 reaction energies subsets from GMTKN55 (33 reactions in total). "Small" includes 33 reactions sampled from "Small54" set including the ISO34 and PArel reaction energies subsets from GMTKN55 (54 reactions in total). (d): Boxplots for the sizes of molecules in the "Big" and "Small54" sets.

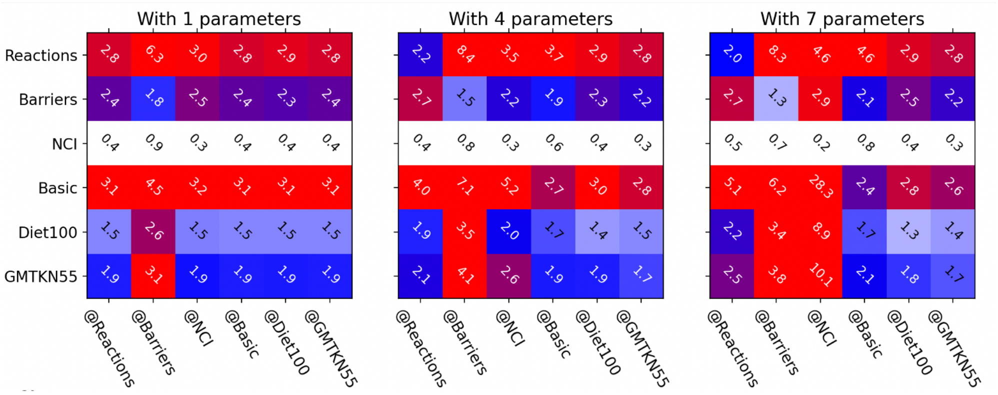

FIG. S12: Same as Fig. 1(c), but with $\mathrm{MAD}_{A@B}$ (kcal/mol) shown in place of $T_{A@B}$.

S7. FURTHER DETAILS ON THE RESULTS IN FIGURE 2(A)

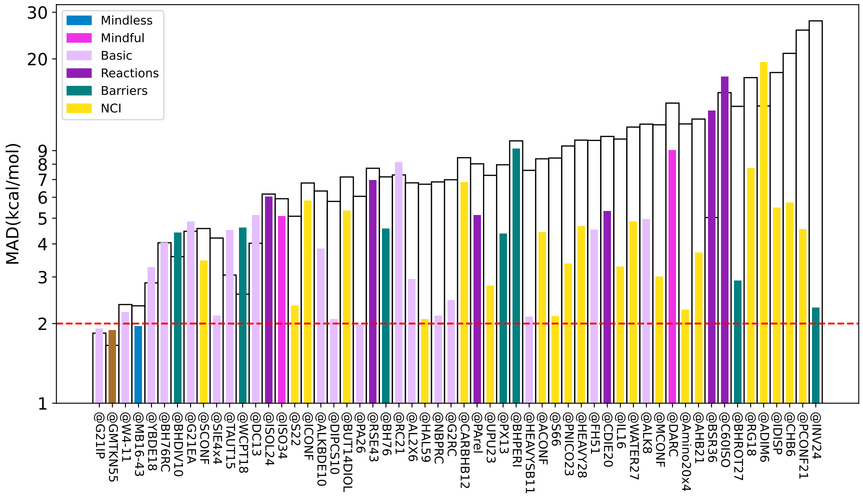

(a) PBE parent

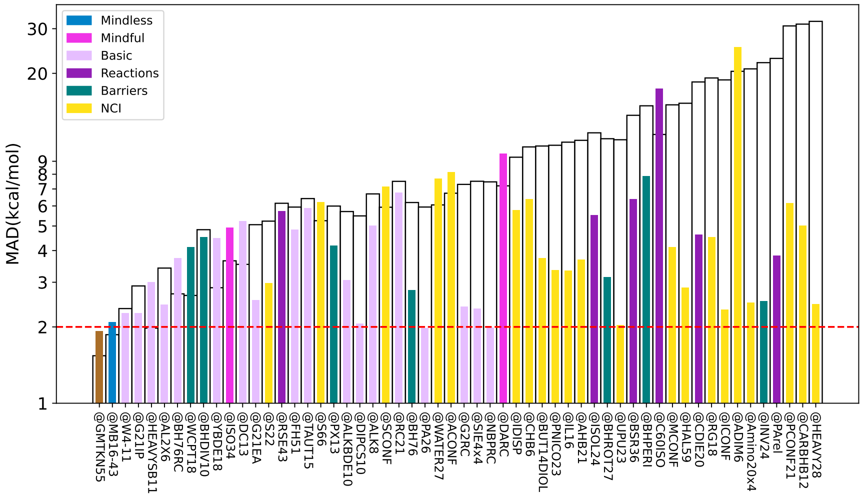

(b) r^{2}SCAN parent

FIG. S13: Same as figure 2(a), but with PBE and r^{2}SCAN parents.

The functional form used in Figure S14 is given by:

$$E_{\mathrm{xc}} = a_1 E_{\mathrm{x}}^{\mathrm{HF}} + a_2 E_{\mathrm{x}}^{\mathrm{LDA}} + a_3 E_{\mathrm{x}}^{\mathrm{B88}} + a_4 E_{\mathrm{c}}^{\mathrm{LDA}} + a_5 E_{\mathrm{c}}^{\mathrm{LYP}} + a_6 E_{\mathrm{x}}^{\mathrm{MP2_{ss}}} + a_7 E_{\mathrm{x}}^{\mathrm{MP2_{os}}} + a_8 E_{\mathrm{c}}^{\mathrm{PBE}} + a_9 E_{\mathrm{c}}^{\mathrm{PBE}} + a_{10} E_{\mathrm{c}}^{\mathrm{r2SCAN}} + a_{11} E_{\mathrm{c}}^{\mathrm{r2SCAN}} \tag{S2}$$

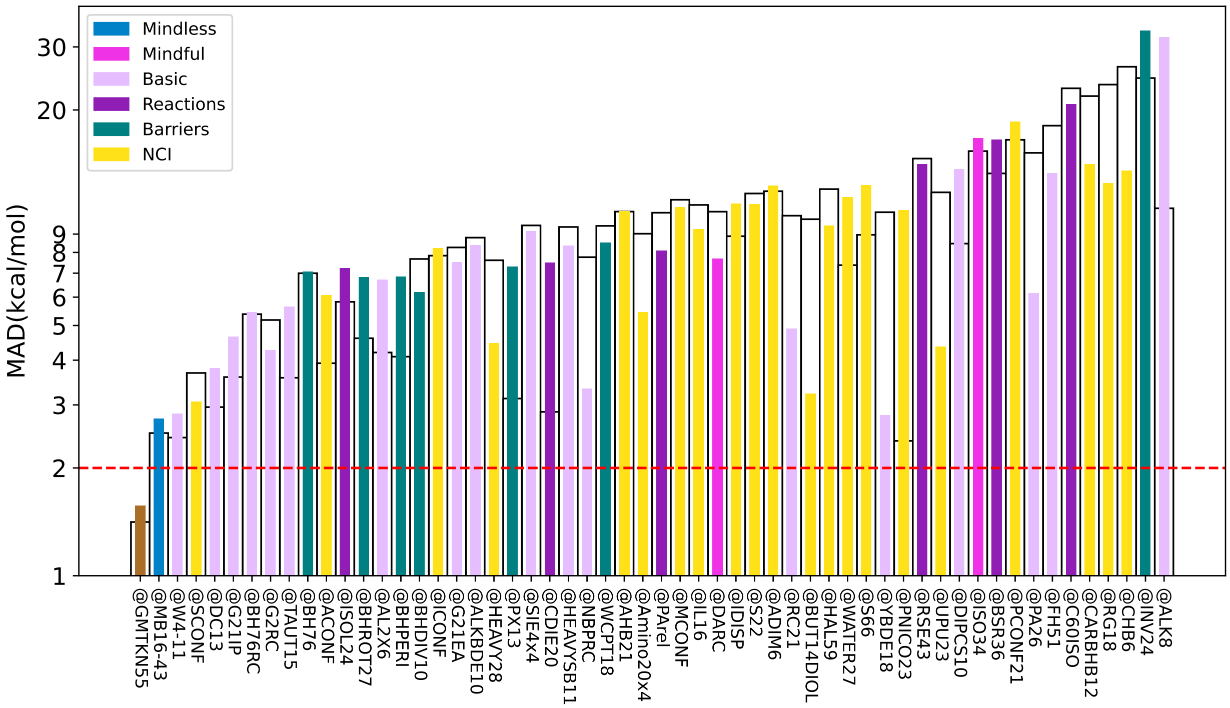

FIG. S14: Same as Figure 2a, but with double hybrids with 9 (DH9) and 11 (DH11) parameters. For DH11, we used functional form of Eq. S2. For DH9 we used the same form, but with a_{10} and a_{11} set to zero.

S8. DETAILS ON THE MINDFUL VS. MINDLESS ANALYSIS

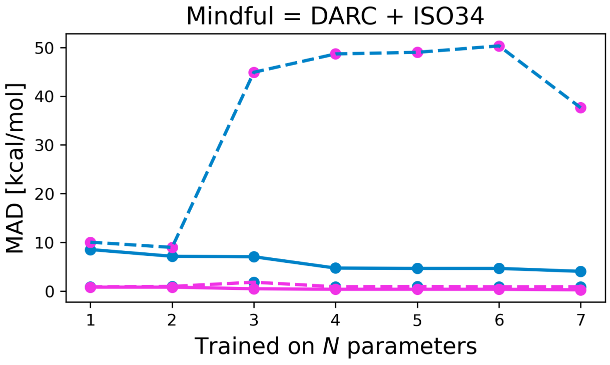

(a)

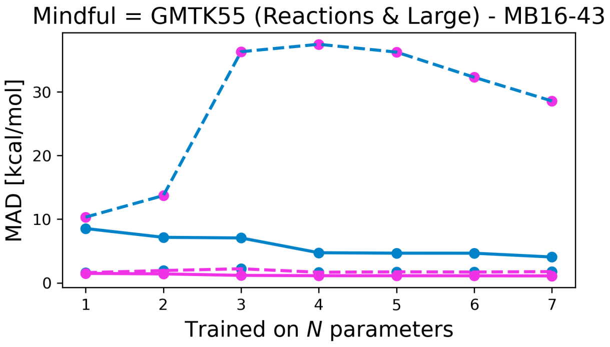

(b)

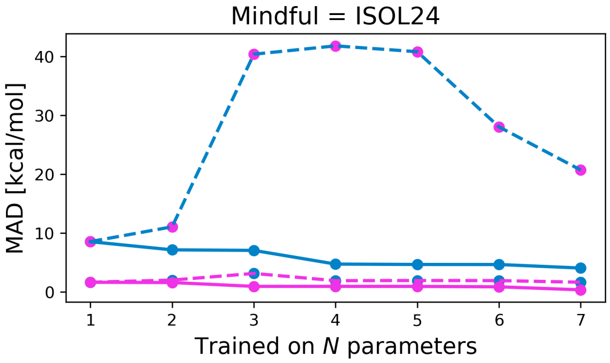

(c)

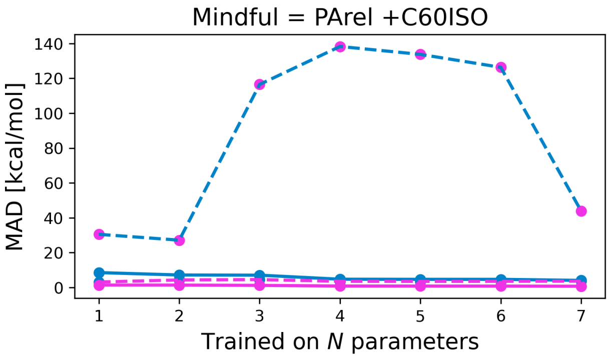

(d)

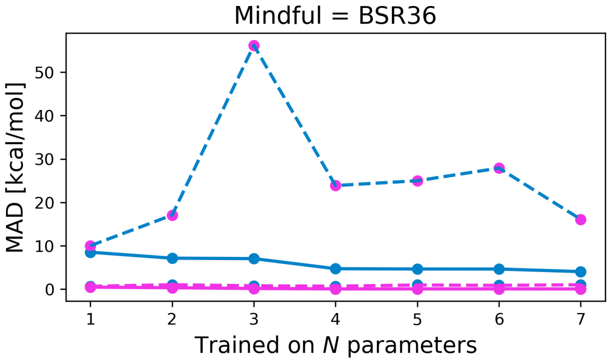

(e)

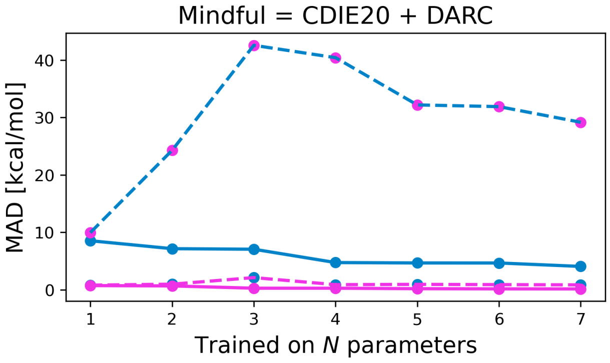

(f)

FIG. S15: Same as Figure 2(b), but with varying the "Mindful" dataset.

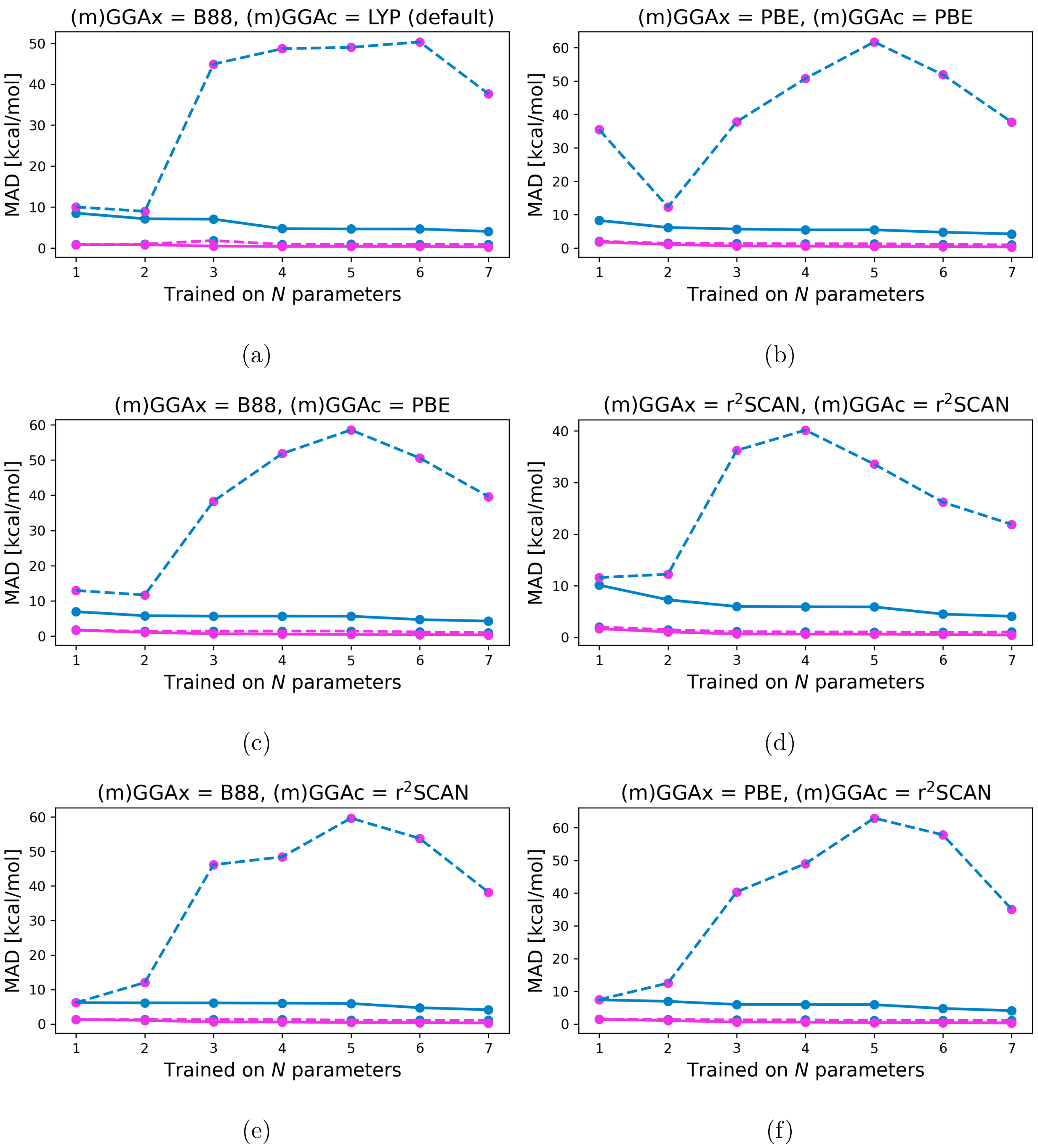

FIG. S16: Same as Figure 2(b), but with varying the (m)GGA parts in double hybrid forms.

S9. FURTHER DETAILS ON THE RESULTS IN FIGURE 3

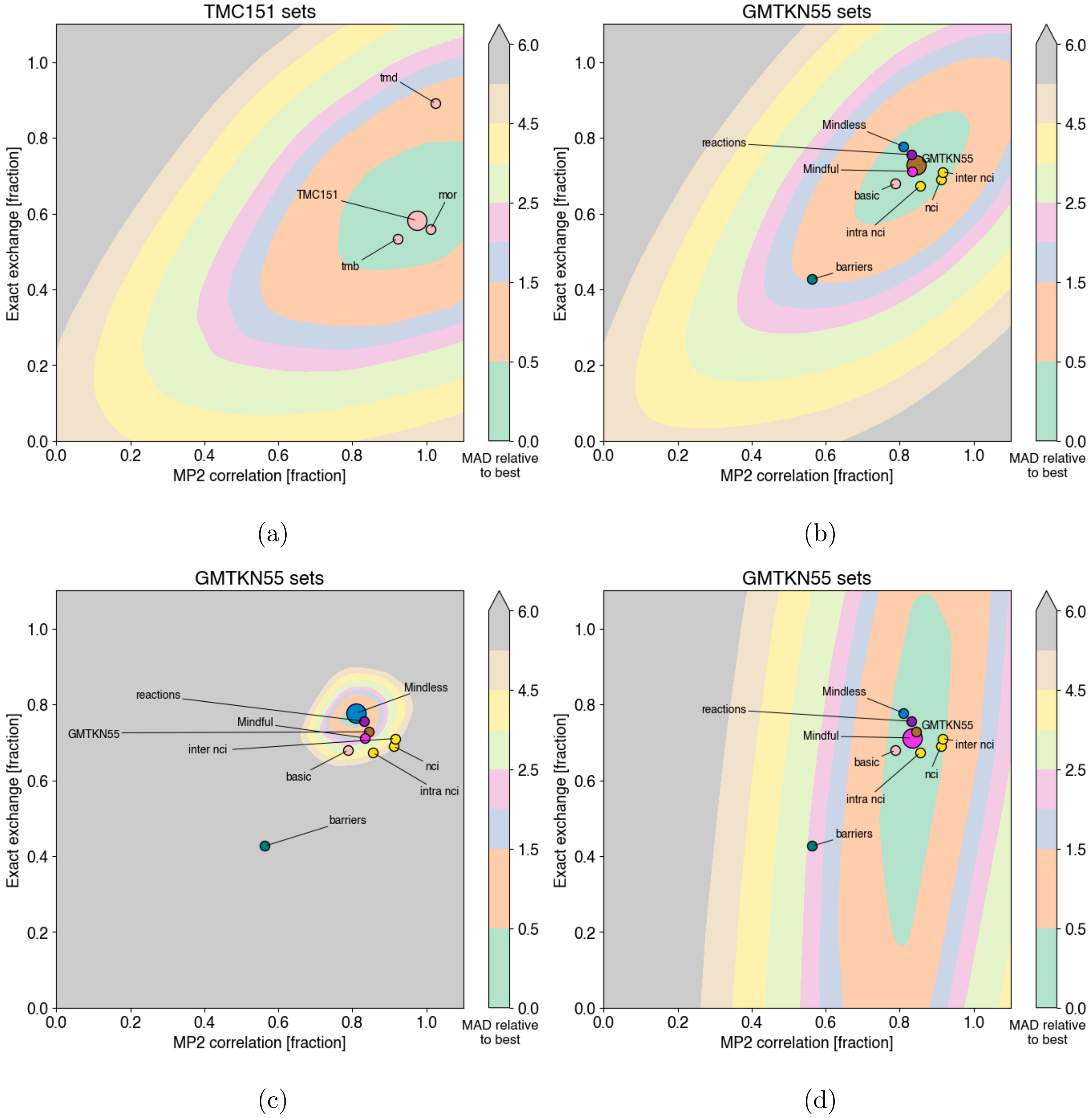

FIG. S17: Same as Figure 3, but with more datasets. 'MAD relative to best' in kcal/mol corresponds to the MAD dataset represented by the largest marker.

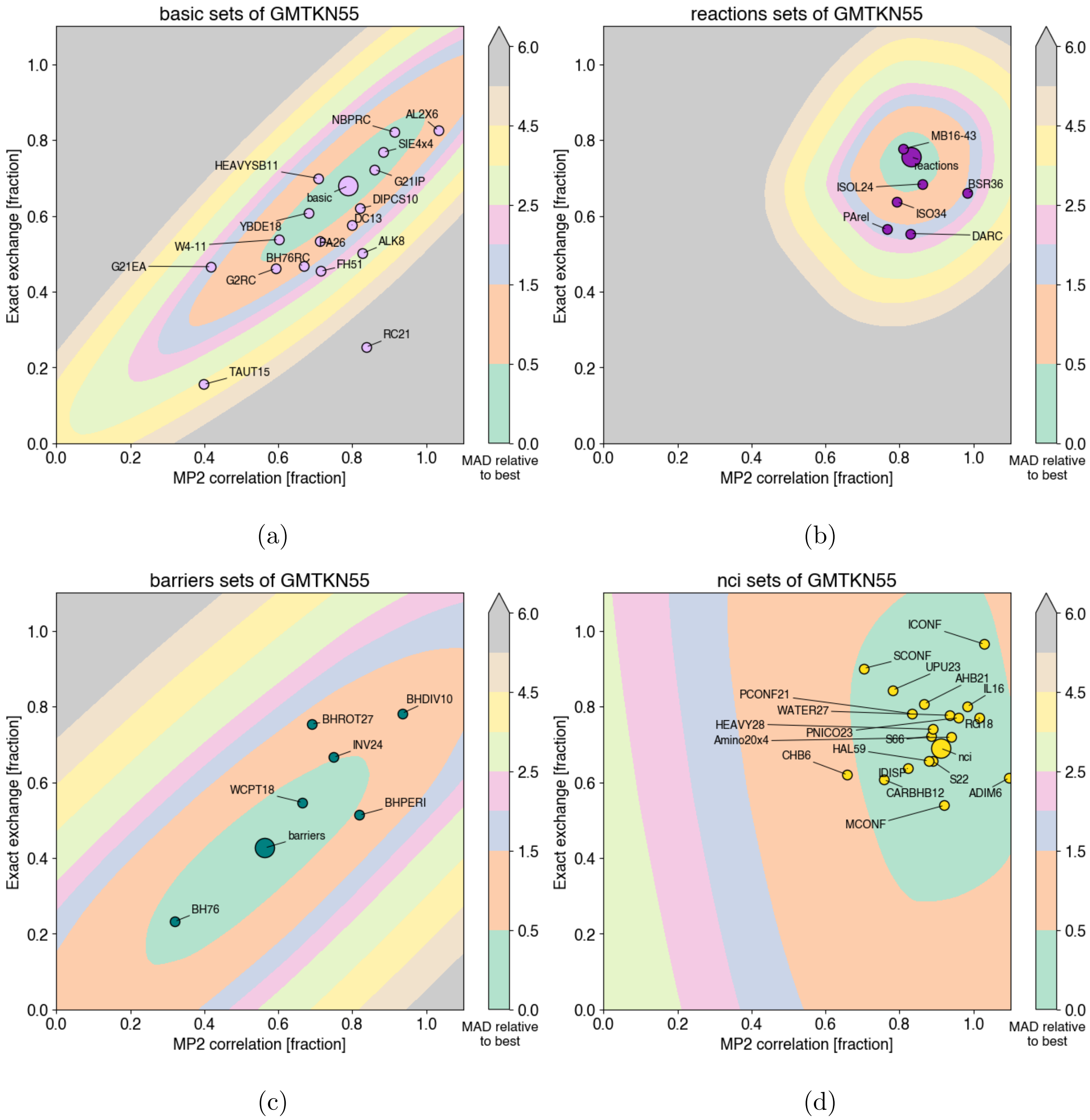

FIG. S18: Same as Figure 3, but with more datasets. 'MAD relative to best' in kcal/mol corresponds to the MAD dataset represented by the largest marker.

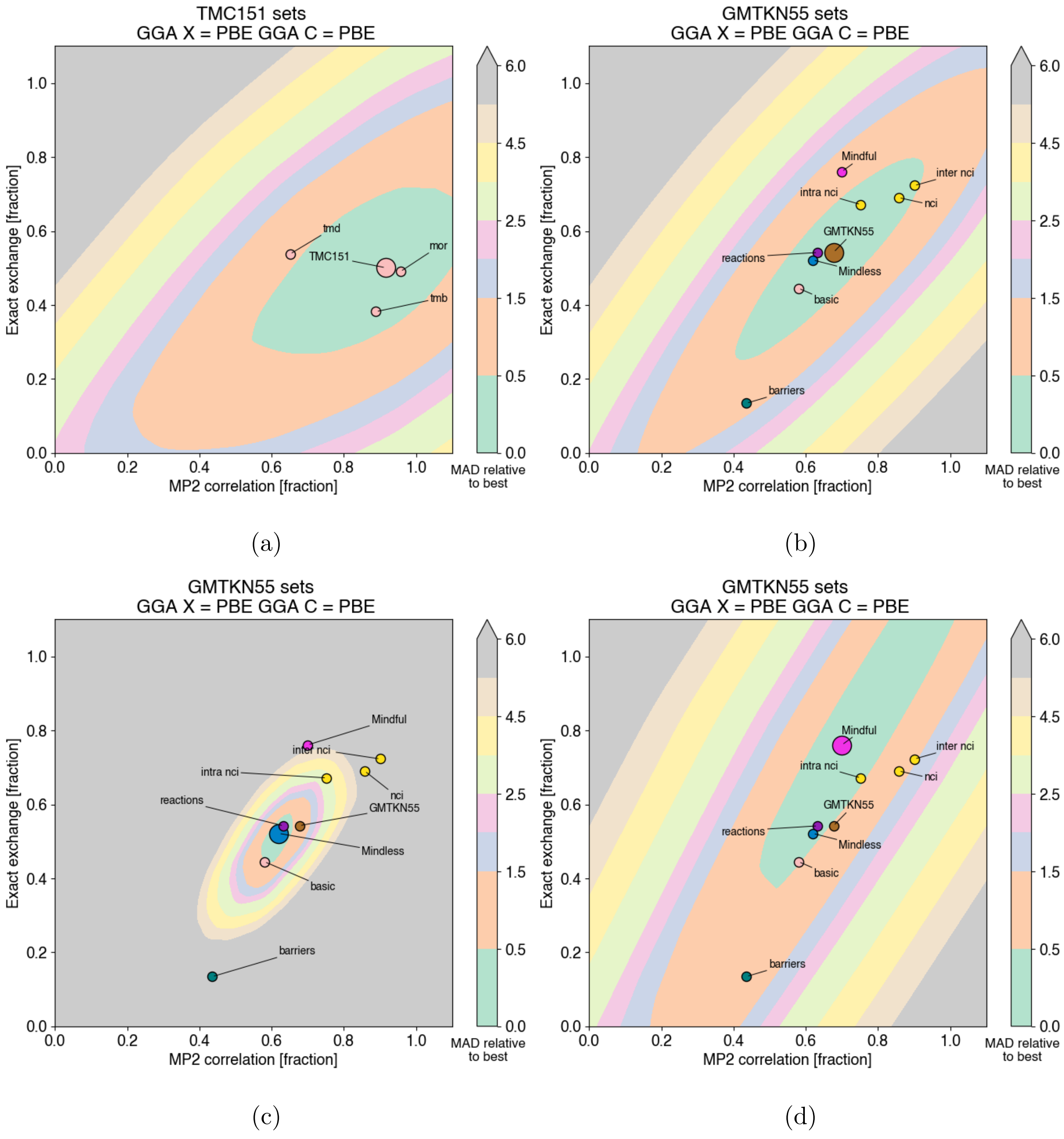

FIG. S19: Same as Figure S17, but with a double hybrid functional consisting of different (m)GGA parts. 'MAD relative to best' in kcal/mol corresponds to the MAD dataset represented by the largest marker.

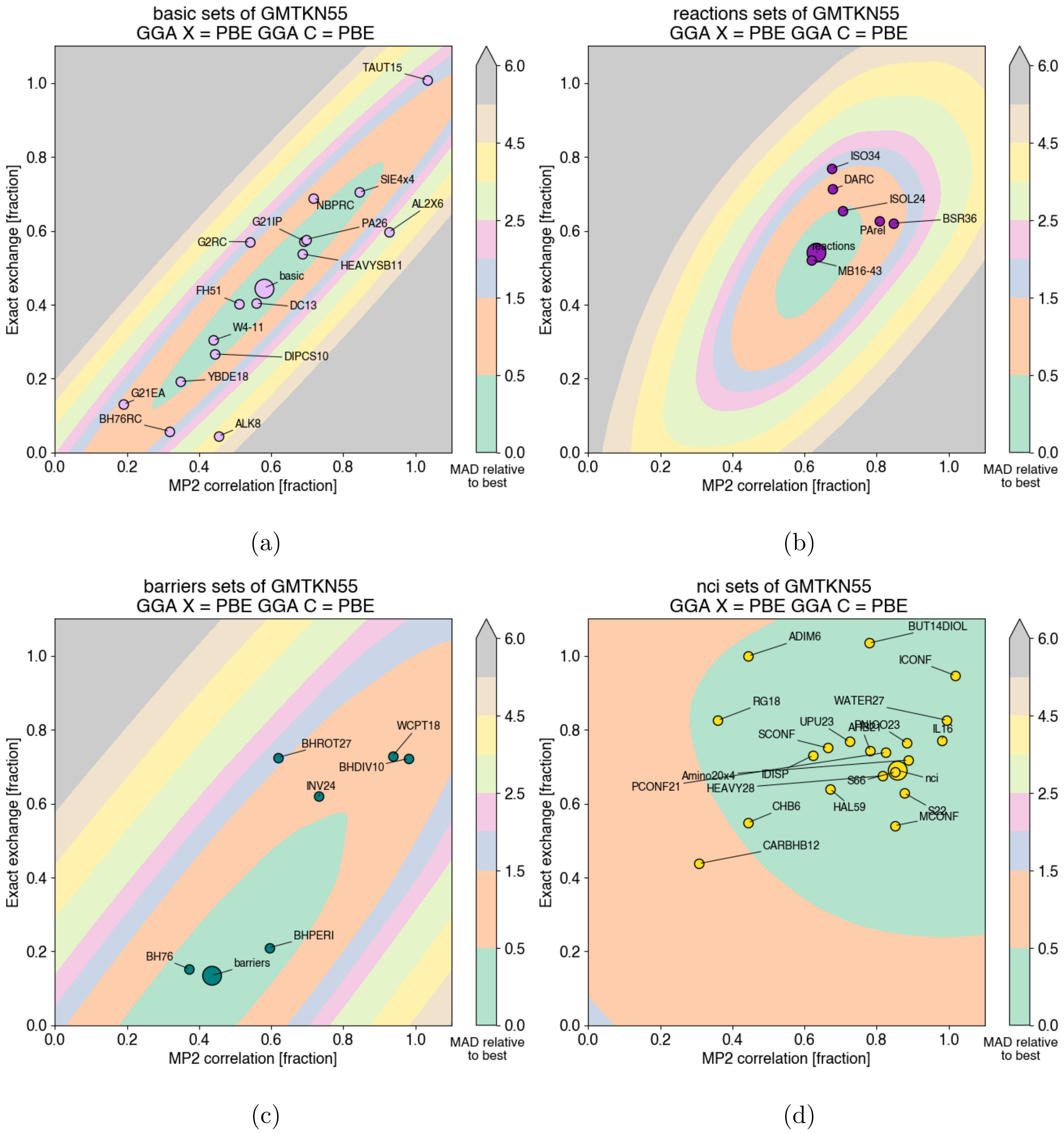

FIG. S20: Same as Figure S18, but with a double hybrid functional consisting of different (m)GGA parts. 'MAD relative to best' in kcal/mol corresponds to the MAD dataset represented by the largest marker.

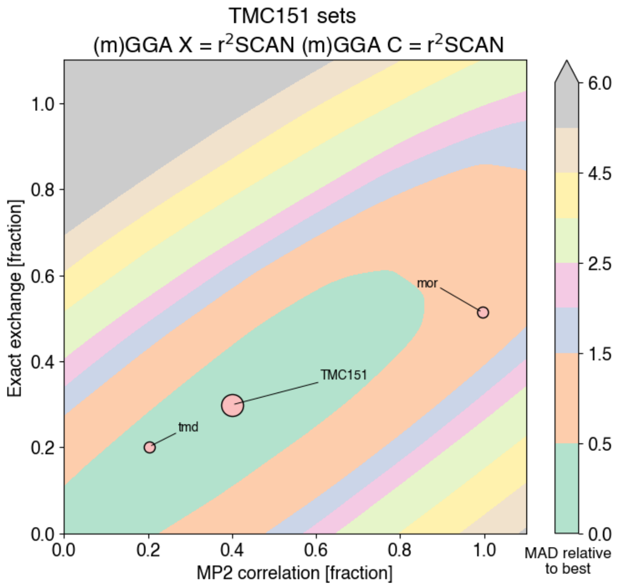

(a)

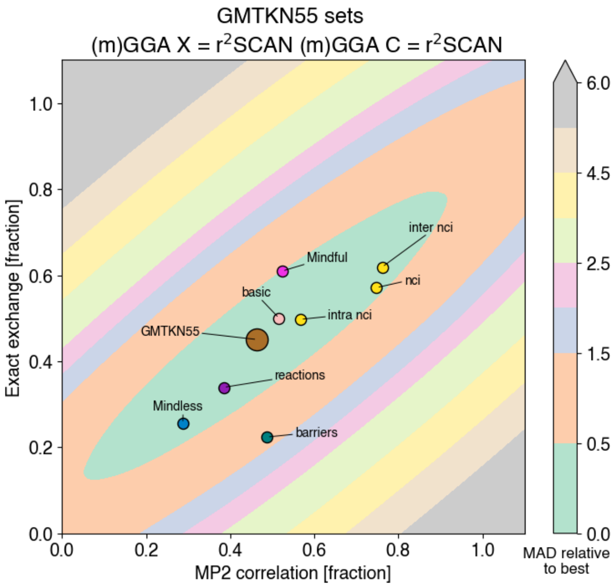

(b)

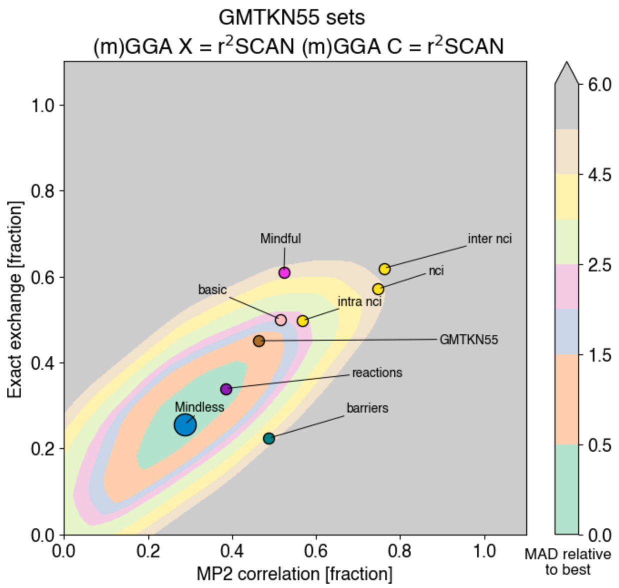

(c)

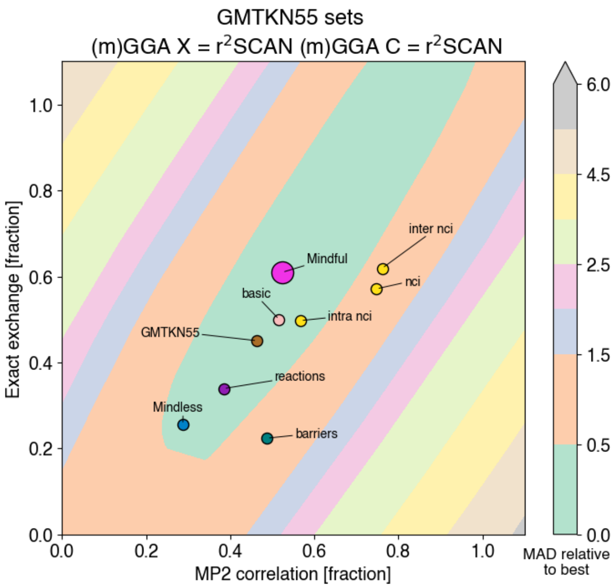

(d)

FIG. S21: Same as Figure S17, but with a double hybrid functional consisting of different (m)GGA parts. 'MAD relative to best' in kcal/mol corresponds to the MAD dataset represented by the largest marker.

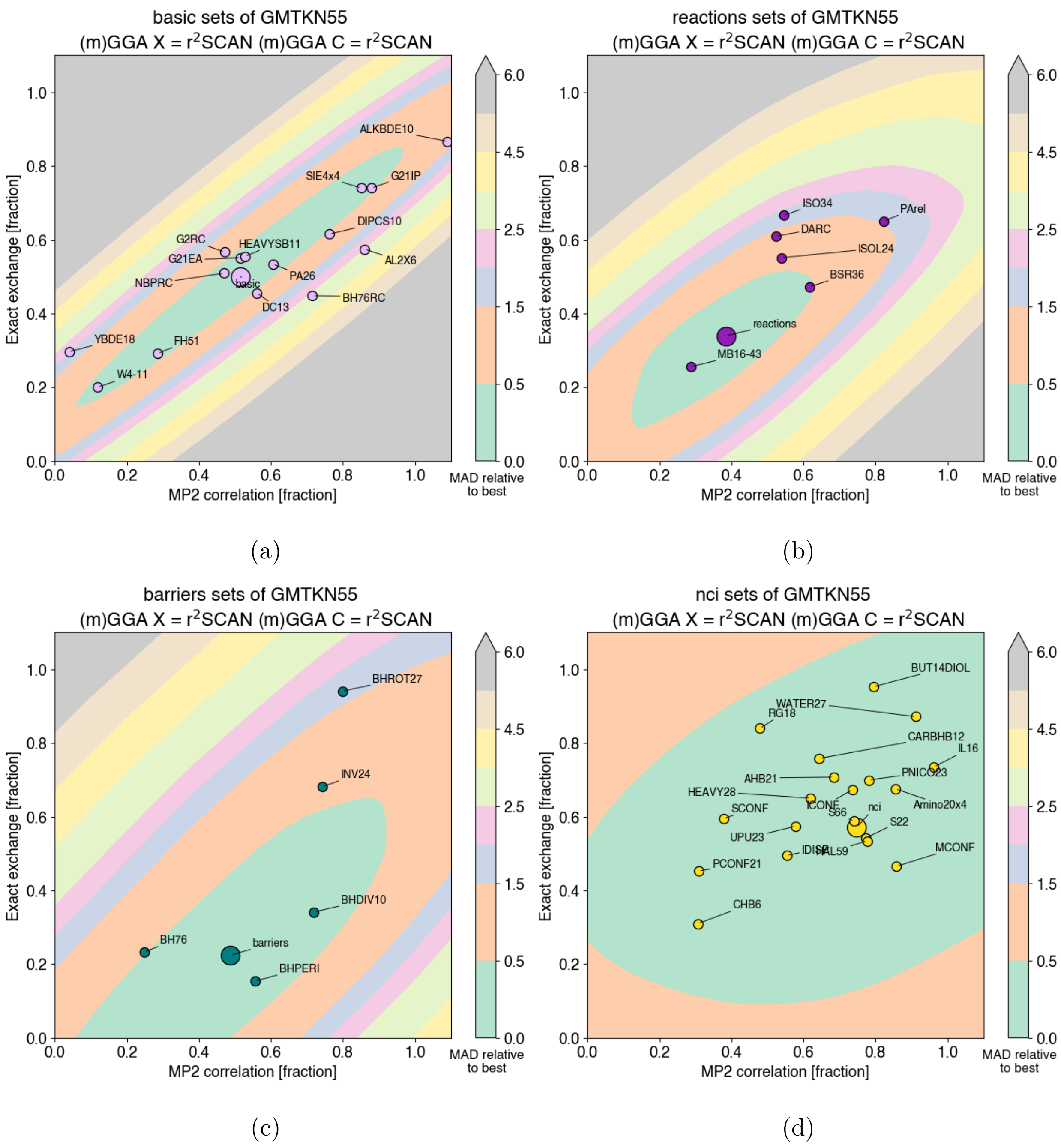

FIG. S22: Same as Figure S18, but with a double hybrid functional consisting of different (m)GGA parts. 'MAD relative to best' in kcal/mol corresponds to the MAD dataset represented by the largest marker.

S10. ADDITIONAL DETAILS FOR TM VS ORGANIC CHEMISTRY TRANSFERABILITY

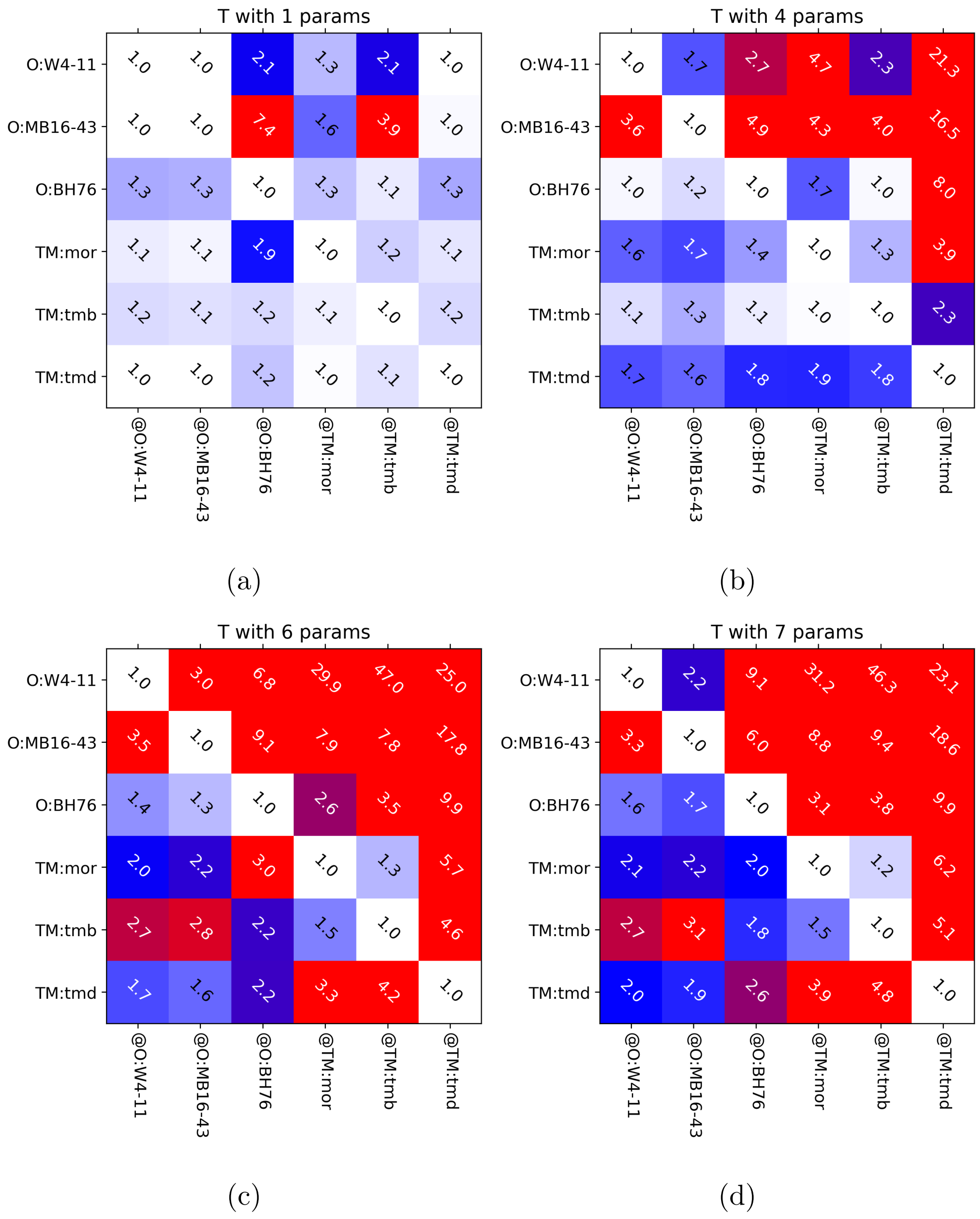

FIG. S23: Transferability matrices for selected TMC151 [”TM” label here] and GMTKN55 [”O” label here] sets. **O@O** and **TM@TM** blocks of the matrices shows the intra-TMC and intra-GMTKN55 transferability. **O@TM** blocks shows how transition metal sets transfer to organic ones. **TM@O** blocks shows how organic sets transfer to transition metal ones. η set to 1kcal/mol as the denominator when train only a single subset of GMTKN55 can get very small.

S11. ADDITIONAL RESULTS FOR SIE4X4 SET

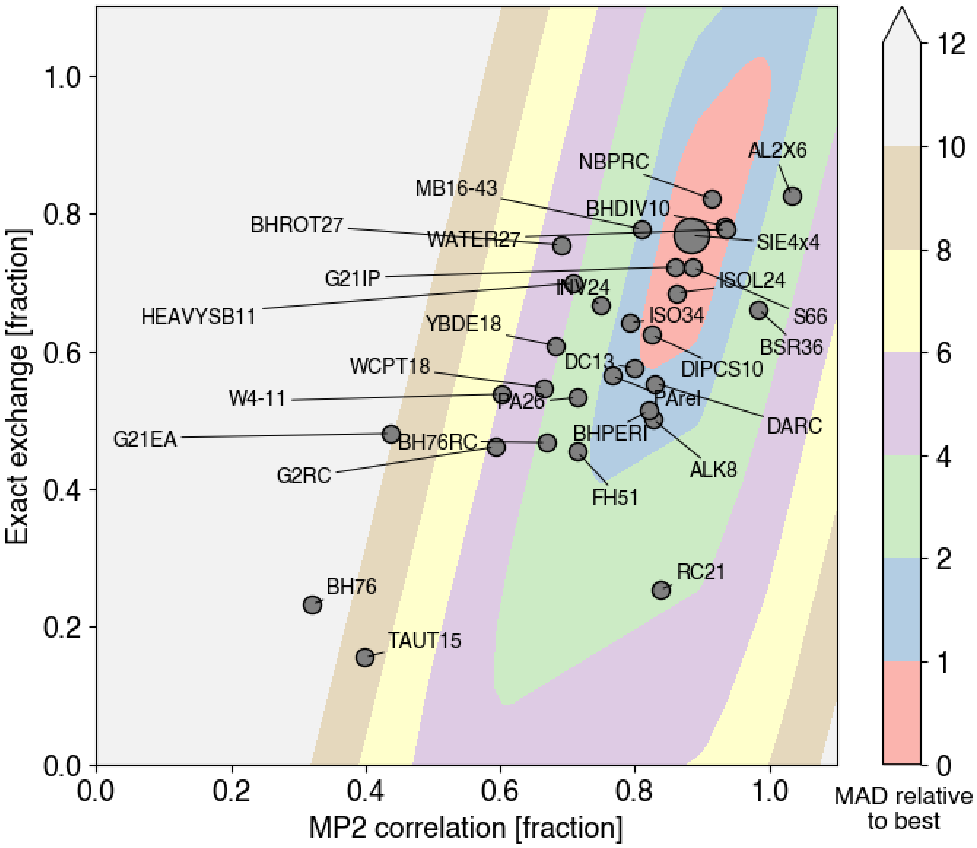

FIG. S24: Optimal values for the two-parameter model, XYG_2 (markers) for selected GMTKN55 sets. Also shows the MAD (contours, kcal/mol) of SIE4x4 set as a function of the two parameters, relative to the optimal value.

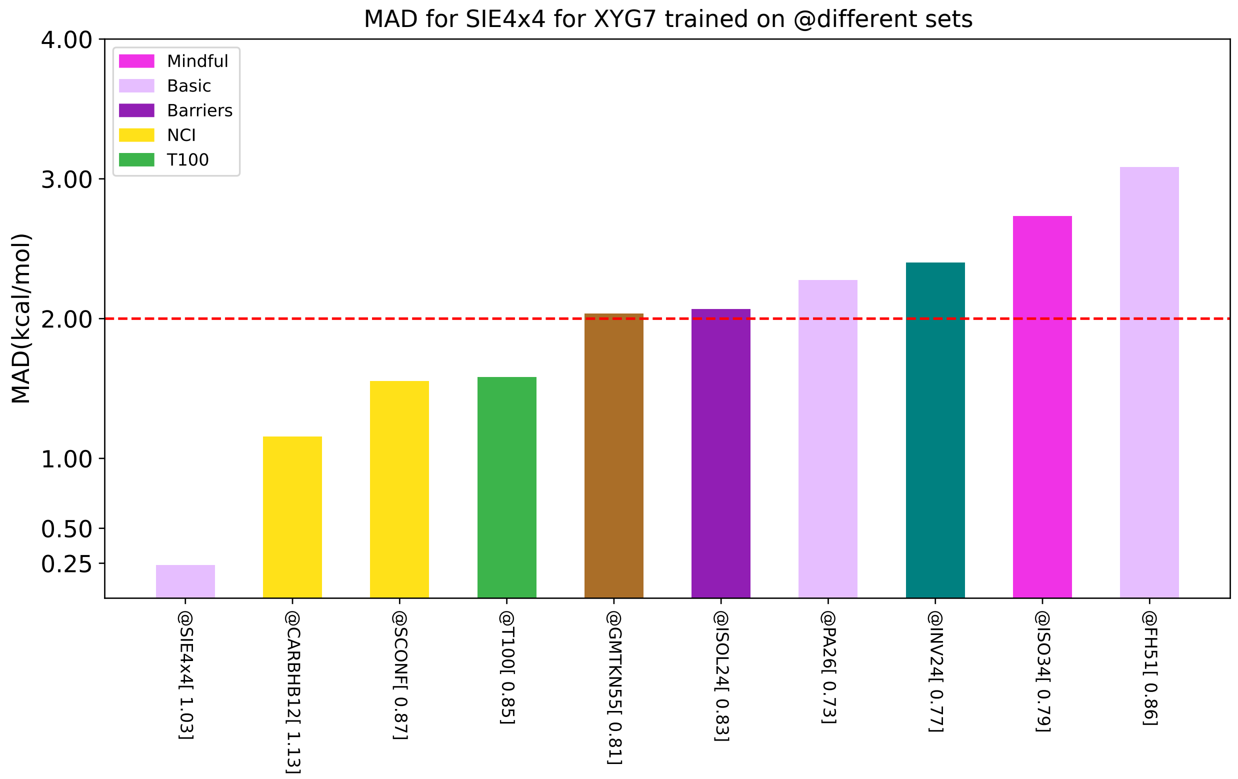

FIG. S25: Mean absolute deviation (MAD) for SIE4x4@set, where set is a subset of GMTKN55 for XYG_7. 10 sets that give lowest SIE4x4@set MAD are shown. Below each bar, the name of @set is shown together with the fraction of exact exchange in XYG_7 train on each individual set. For all shown bars (cases where SIE4x4@set MAD is the lowest), fraction of exact exchange is always greater than 73 percent.

S12. ADDITIONAL RESULTS FOR THE ACCURACY OF @T100-BASED FUNCTIONALS

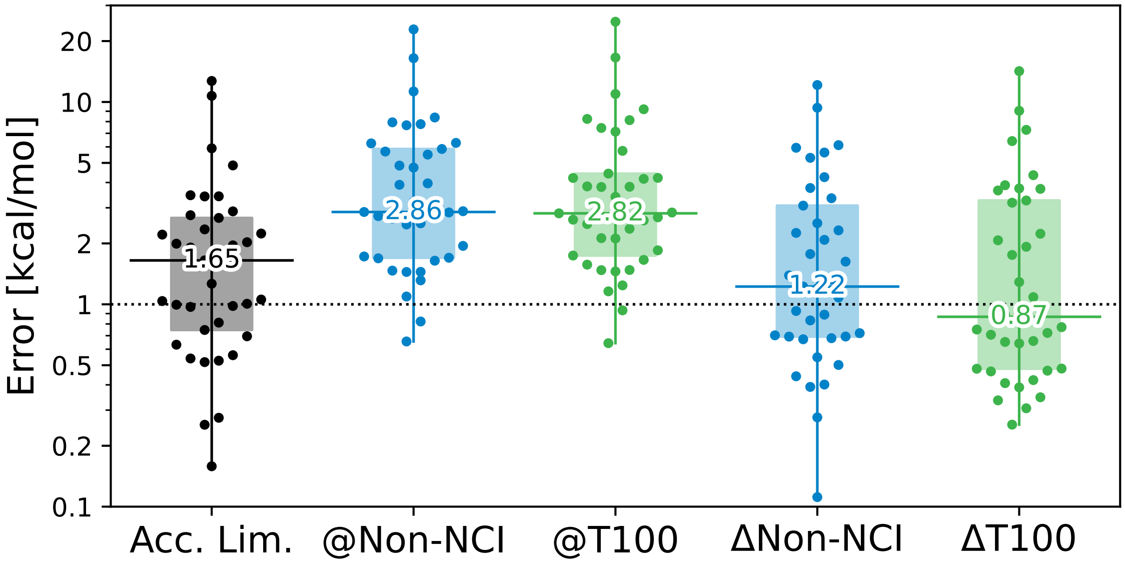

FIG. S26: Transferability energy (same as Fig. 4) with the B3LYP functional form (still with HF orbitals), for the non-NCI subsets of GMTKN55. The black "Acc. Limit" displays MADs for each non-NCI subset using "@Self" for training. Comparisons are drawn with results trained on the complete non-NCI GMTKN55 set (blue bar) and the T100 set (green bar). The final two Δ bars represent the difference between the full dataset and "@Self" training (blue minus black) and T100 and "@Self" training (green minus black). The non-NCI portion of GMTKN55 is selected due to B3LYP's inability (lacking MP2 admixture or dispersion corrections) to capture, dispersion interactions, which are crucial for simulating NCIs.

S13. DATASETS DESCRIPTION

Alias	Name	Description	Reference
n/a.	GMTKN55	Database for general main group chemistry[c]	1
Org.	GMTKN55 minus NCI	GMTKN55 with noncovalent interaction sets removed	1
Mindless	MB16-43	Mindless set with decomposition energies of artificial molecules	1 and 2
Mindful	DARC[a] + ISO34[b]	Cheical intuition-based counterpart of Mindless, combining DARC and ISO34 sets	1
Org. Difficult	P30-5 subset of GMTKN55	Difficult subset of GMTKN55	3
Diet100	Diet100	Gould's statistical representation of GMTKN55 with 100 reactions	4
n/a	G21IP	GMTKN55 subset with adiabatic ionisation potentials	1
Barriers	Barriers - GMTKN55	GMTKN55 subset with barrier heights combining BH76, BHPERI, BHDIV10, INV24, BHROT27, PX13, WCPT18 sets	1
Reactions	Reactions - GMTKN55	GMTKN55 subset with MB16-43, DARC, RSE43, BSR36, CDIE20, ISO34, PArel, C60ISO, ISOL24 sets	1
Org. X	Subset of GMTKN55	A subset from GMTKN55, e.g., Org. Barriers.	1
TM	TMC151	Transition metal chemistry set with 151 reactions	5
n/a	MOR41	TMC151 subset with 41 closed-shell organometallic reactions	5
n/a	TMD60	TMC151 subset with 60 TM dimer dissociation energies;	5
n/a	TMB50	TMC151 subset with 50 barriers of complexes of secondand third-row transition metals	5
TM Difficult, TMDiff	Difficult Subset of TMC151	Includes TMD60 + challenging reactions from TMB50 and MOR41	5
TM+Org.	Org + TMC151	Combines Org and TMC151	1 and 5
T100	Subset of GMTKN55+TMC151	Based on transferable diversity principles	this work

TABLE S2: Summary of used chemical datasets and their descriptions. Notes: [a]Reaction energies of Diels–Alder reactions. [b]Isomerisation energies of small and medium-sized organic molecules. [c]Main group thermochemistry, kinetics, and noncovalent interactions.

REFERENCES

[1]L. Goerigk, A. Hansen, C. Bauer, S. Ehrlich, A. Najibi, and S. Grimme, "A look at the density functional theory zoo with the advanced GMTKN55 database for general main group thermochemistry, kinetics and noncovalent interactions," Phys. Chem. Chem. Phys. **19**, 32184–32215 (2017).

[2]M. Korth and S. Grimme, ""mindless" DFT benchmarking," J. Chem. Theory Comput. **5**, 993–1003 (2009).

[3]T. Gould and S. G. Dale, "Poisoning density functional theory with benchmark sets of difficult systems," Phys. Chem. Chem. Phys. **24**, 6398–6403 (2022).

[4]T. Gould, "'diet GMTKN55' offers accelerated benchmarking through a representative subset approach," Phys. Chem. Chem. Phys. **20**, 27735–27739 (2018).

[5]B. Chan, P. M. W. Gill, and M. Kimura, "Assessment of DFT methods for transition metals with the TMC151 compilation of data sets and comparison with accuracies for main-group chemistry," J. Chem. Theory Comput. **15**, 3610–3622 (2019).
